# Gut microbiome-metabolome interactions predict host condition

**DOI:** 10.1186/s40168-023-01737-1

**Published:** 2024-02-10

**Authors:** Oshrit Shtossel, Omry Koren, Iris Shai, Ehud Rinott, Yoram Louzoun

**Affiliations:** 1 Department of Mathematics, Bar-Ilan University, Ramat Gan, 52900 Israel; 2 The Azrieli Faculty of Medicine, Bar-Ilan University, Safed, Israel; 3 Faculty of Health Sciences, Ben-Gurion University of the Negev, Beer-Sheva, Israel

## Abstract

**Background:**

The effect of microbes on their human host is often mediated through changes in metabolite concentrations. As such, multiple tools have been proposed to predict metabolite concentrations from microbial taxa frequencies. Such tools typically fail to capture the dependence of the microbiome-metabolite relation on the environment.

**Results:**

We propose to treat the microbiome-metabolome relation as the equilibrium of a complex interaction and to relate the host condition to a latent representation of the interaction between the log concentration of the metabolome and the log frequencies of the microbiome. We develop LOCATE (Latent variables Of miCrobiome And meTabolites rElations), a machine learning tool to predict the metabolite concentration from the microbiome composition and produce a latent representation of the interaction. This representation is then used to predict the host condition.

LOCATE’s accuracy in predicting the metabolome is higher than all current predictors. The metabolite concentration prediction accuracy significantly decreases cross datasets, and cross conditions, especially in 16S data.

LOCATE’s latent representation predicts the host condition better than either the microbiome or the metabolome. This representation is strongly correlated with host demographics. A significant improvement in accuracy (0.793 vs. 0.724 average accuracy) is obtained even with a small number of metabolite samples ($\sim 50$).

**Conclusion:**

These results suggest that a latent representation of the microbiome-metabolome interaction leads to a better association with the host condition than any of the two separated or the simple combination of the two.

Video Abstract

**Supplementary Information:**

The online version contains supplementary material available at 10.1186/s40168-023-01737-1.

## Introduction

The human gut microbial composition is associated with multiple aspects of human health [1–6]. The microbiome is associated with human health, either directly through the effect of microbes on disease [7–10], or indirectly through interaction with different systems of the human host [9–13]. However, the most extensive interaction with the host is through metabolite consumption and production [14–17] with short-chain fatty acids (SCFAs, see Supplementary material Acronym Table S6) such as butyrate, acetate, and propionate, the end product of gut microbiome fermentation, being some of the most studied metabolites [16, 18]. SCFAs have been shown to have a role in regulating the immune response and gut barrier function, gut cell proliferation and differentiation, regulation of gut endocrine functions, and even in gut brain axis communication [19, 20]. The relation between metabolites and microbes is bi-directional, with each affecting the frequency/concentration of the other. However, typically, the prediction was from the microbiome to the metabolites [21, 22] and not vice-versa. Indeed, metabolites have been shown to be affected by heritable, gut microbiome, by lifestyle choices such as smoking, or by diet [23].

Both microbiome and metabolome have been associated with the host condition through either correlations or predictions [22], often in conjunction with additional meta-data, such as age, gender, or diet [24–31]. Such prediction typically requires ML models, including deep neural networks (DNNs) and convolutional neural networks (CNNs) [28–31]. However, inferring the human condition based on either microbiome or metabolome separately suffers from several limitations. The limitations of microbiome-based ML include among others little knowledge about the interaction between different members of the microbial community or with the host, and the absence of mechanistic understanding of the relation between the microbiome and health or disease [32–34]. Microbiome-based ML is also often plagued by a low prediction accuracy vs. other sources of information, such as metabolites [25–27].

While metabolome studies have become increasingly used in characterizing emerging properties of the metabolome and in relating metabolomic change to host pathological states [35–45], metabolome-based ML also has its limitations: (1) high cost; (2) extremely high dimension of input (i.e., number of different metabolites vs. the number of samples), especially in untargeted studies [25]; (3) a large number of unknown metabolites that have a molecular composition, but no known function [46, 47]; (4) large variability of nomenclature and experimental protocols among different studies [48, 49].

We here propose that the microbiome-metabolome combination can be used to produce a non-linear intermediate latent representation (marked as *Z* all along the manuscript) that is closely associated with the host conditions and can be used to predict them. We then propose an algorithm to compute this representation and show that it simultaneously improves the prediction of metabolome (log concentration) from the microbiome (log composition) and the prediction of the host condition. We denote this algorithm as LOCATE (Metabolites prediction by Latent variables Of miCrobiome And meTabolites rElations).

**Fig. 1:**
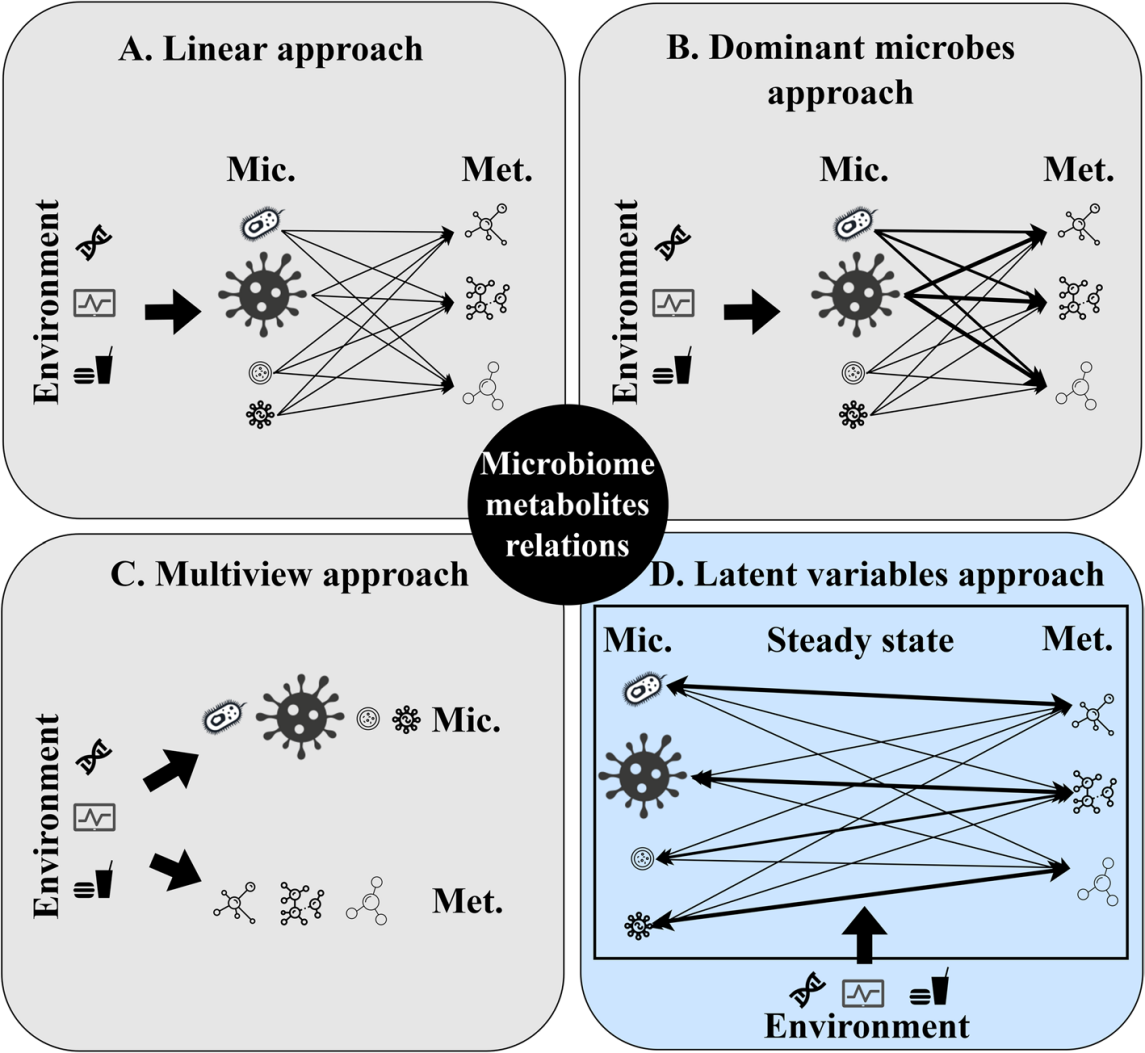
Different approaches to microbiome-metabolites relations. **A** The linear approach assumes each microbe produces some metabolites, and the metabolite concentration is a positive linear combination of the microbe concentration. The environmental/host effect if any is only through the microbiome (gray). **B** The dominant microbes approach assumes that given the dominance of a small number of microbe species composing the vast majority of microbes in the gut, the concentration of each metabolite is determined by the most frequent microbes (gray). **C** Multiview assumes the environment directly affects the metabolites and microbiome separately (gray). **D** The latent variables approach assumes that the microbiome and metabolome represent the steady state of a bi-directional interaction. In such a case, the equilibrium can differ between hosts. However, it may be represented by some combined latent representation (blue). In all plots, microbiome taxa are symbolized by bacteria icons of various sizes, reflecting their frequencies. “Mic.” denotes the microbiome, and “Met.” stands for metabolites. The arrows denote interactions, with arrow thickness indicating the strength of the interaction and the arrow direction indicating the direction of the interaction

To understand this claim, one can contrast simple models of microbiome-metabolome relations:

A) *Linear model*: This model is focused on metabolite production. Each microbe produces metabolites, and the metabolite concentration is a positive linear combination of these microbe productions. The metabolite concentration can thus be described by a non-negative factorization of the microbe frequencies, which would basically capture the contribution of each microbe to each metabolite studied. Some studies propose qualitative relations, where each microbe is associated with high or low values of a metabolite [50–52]. Those are mostly based on biological relations of production and consumption. Other studies propose quantitative relations. Some of those are reference-based, such as *Predicted Relative Metabolomic Turnover* (PRMT), *MIMOSA* (Model-based Integration of Metabolite Observations and Species Abundances), and *Mangosteen* [53–56], while others are model-based, such as *MelonnPan* [57] (Fig. 1A).

B) *Dominant microbes model*: An alternative hypothesis would be that given the dominance of a small number of microbe species composing the vast majority of microbes in the gut, the concentration of each metabolite is determined by the most frequent microbe. This conceptualization translates into models that relate one main microbe (or set of genetically similar microbes) to each metabolite (Fig. 1B). While this is not implemented in any quantitative model, this is the assumption underlying most qualitative arguments suggesting that changing a dominant microbe would change the metabolite concentration.

C)*Multiview model*: Contrary to the 2 former approaches, which assume the microbiome and metabolites interactions are direct and the environment affects the situation via the microbiome only [21], this model assumes the microbiome and metabolites are both affected by the environment. Therefore, the conditions of the samples (which determine the environment for the microbiome and metabolome) can be estimated from the microbiome and metabolites by using multi-omics approaches, such as *Multiview* and *IntegratedLearner* [58, 59]. Despite its multi-faceted approach, this model falls short of creating a learnable connection between the microbiome and metabolites (Fig. 1C).

D) *Latent variables model*: We here follow a model where the observed frequencies of microbes and concentrations of metabolites represent the steady state of complex bi-directional interactions [22, 23]. In such a case, the effect of a microbe on metabolites is not linearly or positively correlated with its frequency. Moreover, the equilibrium is affected by the environment (e.g., heritability, lifestyle choices, and diet) and differs between hosts. While this complicates modeling the relationship between metabolites and microbiome, it produces a latent representation of the relation. This representation can then be directly associated with the environment (Fig. 1D). We propose that this approach is indeed better than the ones above for predicting the relation between microbes, metabolites, and the host condition.

There are also more heuristic ML-based models that do not fit clearly into these categories, such as *MiMeNet* [60], and encoder-decoder-based models, such as *SparseNED* [27], *mNODE* [61], and the model proposed by Khajeh et al*.* [25] that showed that autoencoders of microbiome and metabolome can be used for IBD prediction.

## Related work

The current analysis has two main stages: First, the prediction of the metabolite concentration from the microbe frequencies and the resulting latent representation; second, the prediction of the host condition is based on this representation. Multiple models were proposed for both stages (for a summary see Supplementary material Table S1).

### Linear models

Different approaches have been proposed in recent years to link the microbiome composition with metabolomic data. One strategy relies on the creation of a connection network linking a given gene/amplicon sequence variant (ASV)/taxon to pathways and compounds in a database. These linkages are used to infer molecular compound identities from the genetic composition of the microbial community. Most methods are descriptive [62–70]. However, there are some quantitative methods. Such methods include predicted relative metabolomic turnover (PRMT) to predict metabolites from a coastal marine metagenomics dataset, showing a clear correlation between the predicted metabolites and environmental factors [53]. MIMOSA was later developed to predict metabolic potential in a given microbial community and to identify the microbial taxa most associated with the synthesis/consumption of key metabolites [54, 55]. Both methods rely on a reaction network and are limited to the KEGG database. A similar method to predict metabolites directly is Mangosteen, a metabolome prediction pipeline dependent upon relationships between KEGG/BioCyc reactions and the molecular compounds directly associated with those reactions [56]. All the above methods are reference-based, and as such rely heavily on the completeness and accuracy of the database query. MelonnPan uses ML to predict metabolomic potential scores, which represent the relative capacity of the community in a given sample to generate or deplete each metabolite. MelonnPan has good accuracy on a specific IBD dataset [57].

### Different ML-based models

Similar to MelonnPan, MiMeNet is an MLPNN (multiple-layer perceptron neural network) model that is composed of multiple fully connected hidden layers. They further define well-predicted metabolites [60]. Various methods adopt the encoder-decoder paradigm, for example, SparseNED—a sparse one-layer neural encoder-decoder network predicts metabolite abundances from microbe abundances [27] and Khajeh et al. multi-task autoencoder to extract the latent profiles from the combined microbiome and metabolome data for IBD prediction [25]. A more intricate example is *mNODE *(metabolomic profile predictor using neural ordinary differential equations) [61] which is a deep learning method that combines explicit layers with implicit layers where the states of hidden layers are described by ODEs.

Most current methods are database-specific and cannot be trained on one cohort and tested on another cohort. In other words, they are not transferable. There was a single attempt to perform cross-predictability between datasets in [71] by a random forest regression model. Unfortunately, their success was limited to specific metabolites in specific pairs of datasets.

### Prediction of host condition based on combination of metabolome and microbiome

A distinctive perspective is to use the combined microbiome-metabolome to predict the host condition. Such an approach is adopted by the Multiview model [58], wherein the microbiome and metabolites are treated as distinct perspectives of the host condition (assumed to be the environment affecting the microbiome and metabolome). Multiview uses “Cooperative Learning,” which combines the standard squared-error loss with an “agreement” penalty to encourage the predictions from different data views (microbiome and metabolites) to agree. Another novel approach is the IntegratedLearner [59], which applies Bayesian ensemble methods to consolidate predictions by harnessing information across multiple longitudinal and cross-sectional omics data layers.

## Results

### Relation between microbiome and metabolites is not linear and is dominated by a few taxa

We first tested the linear model. In this model, the relation between metabolites and microbiome is often described through a consumption/production network [50–52, 72, 73]. Such networks are based on three assumptions: (A) Each microbe may consume more than one metabolite or produce different metabolites, and therefore such a network is required. (B) Production and consumption rates are not frequency dependent. As such, one could assume the concentration of the metabolites would depend on a linear combination of their production and consumption by a variety of different microbes. This assumption may fail following non-linear experimental response curves for both microbes and metabolites [74–76] or non-linear consumption/production. (C) The relation production and consumption rate are not affected by external factors or other bacteria [77, 78].

To test the first assumption, we performed an NMF (non-negative matrix factorization) decomposition (see the “Methods” section) of the metabolite non-negative concentrations (relative normalized) over the microbial relative frequencies of 10 paired microbiome-metabolome datasets (5 16S rRNA gene sequencing-based and 5 WGS datasets (see the “Methods” section)). Surprisingly, in most cases (94.9%), a single microbe was associated with more than $80\%$ of the production of a single metabolite, as measured by the NMF coefficient values.

**Fig. 2:**
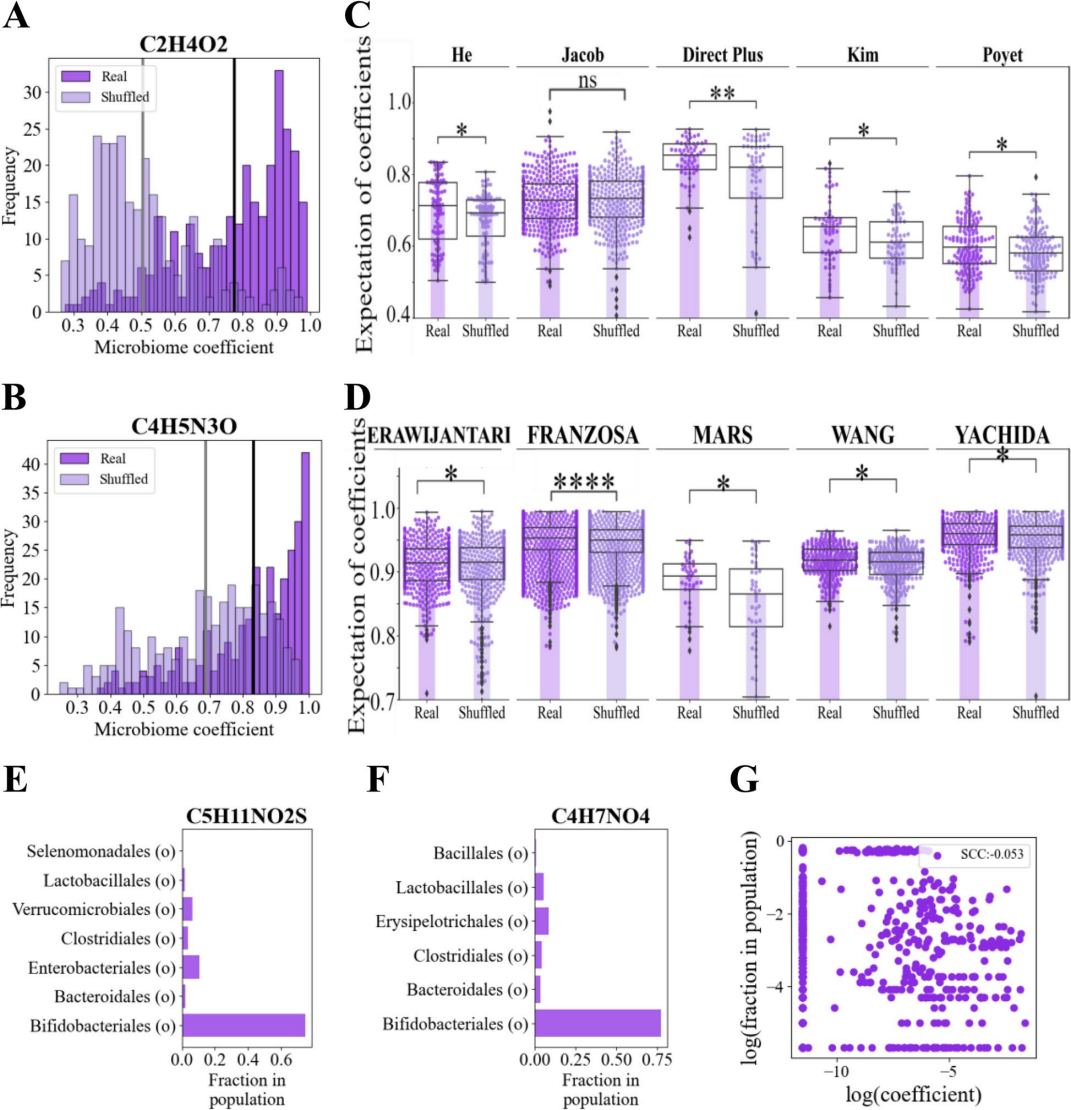
The relation between the microbiome and the metabolites is not linear and is dominated by a few taxa. **A**, **B** Histograms of the coefficients of the NMF model which relates metabolite concentrations and the microbiome frequencies (real in dark purple) and of a random model with the microbes shuffled before the prediction (light purple) of the metabolite C2H4O2 (**A**) and C4H5N3O (**B**). The black line represents the expectation of the real data, and the gray line represents the expectation of the shuffled data. The coefficients of the real model are higher than the coefficients of the shuffled model. Similar results are observed for all the other metabolites as well. **C**, **D** Swarm plots of all the expectations of the relative contribution of the coefficients of each metabolite for all the 16S rRNA gene-based (**C**) and the WGS datasets (**D**). The expectations of the real models are represented in dark purple dots, while the expectations of the shuffled models are in light dots. Bar plots represent the median of each group. A two-sided *t*-test was applied between the two models for each dataset. In all the datasets apart from Jacob, the expectations of the real model are significantly higher (*p*-value $$<0.05)$$ than the expectations of the shuffled model. The stars represent the *p*-values, such that *p-value $$\le 0.05$$, **p-value $$\le 0.01$$, ***p-value $$\le 0.001$$. **E**,** F** Bar plots of the frequency of the microbes associated with the 10 highest coefficients in the NMF models of C5H11NO2S (**E**) and C4H7NO4 (**F**). There are no consistent patterns. For most metabolites, the most frequent order is not the best predictor. **G** Scatter plot of the coefficients in the log NMF model of the taxa with the highest coefficients vs. the logged frequency of the same taxa, with no clear correlation between them

To test that such a skewed effect is not a direct result of the microbiome and metabolome distributions, we compared the real model to the relative contribution of the coefficients of a random parallel model whose microbes are shuffled (Fig. 2A, B, the expectations are in black (real) and gray (shuffled) for the averages of all coefficients’ relative contributions of all metabolites in the He dataset, see Supplementary material Fig. S8) as well as significantly higher coefficient relative contributions expectations for each metabolite (*p*-value $$< 0.05)$$ for all datasets (Fig. 2C, D). As such, the concept of a metabolite-microbe interaction network (linear approach) may fail, and instead, a direct relation between a dominant microbe and a metabolite should be considered (Fig. 2A–D). Thus, the dominant microbe described above is more consistent with the observed interaction than the linear approach.

If we follow the dominant microbes approach, one could presume that the dominant microbes would be the very frequent ones. However, this is not the case. In the 10 most dominant taxa per metabolite (with the highest coefficients), when computing their frequency over the population, rare taxa are often dominant (Fig. 2E, F), the SCC between the microbe’s NMF coefficient and their fraction in the population is typically null $-0.05$ (Fig. 2G). To summarize, neither the linear nor the frequent dominant microbes approach seems to be consistent with the observations. Consequently, we propose to use a non-linear model relating the log microbe frequencies and the log metabolite concentrations (Fig. 2E–G), instead of a linear model or single dominant microbes models.

### Latent representation of a microbiome (LOCATE) can be used to predict metabolites in each dataset separately better than all existing methods

**Fig. 3:**
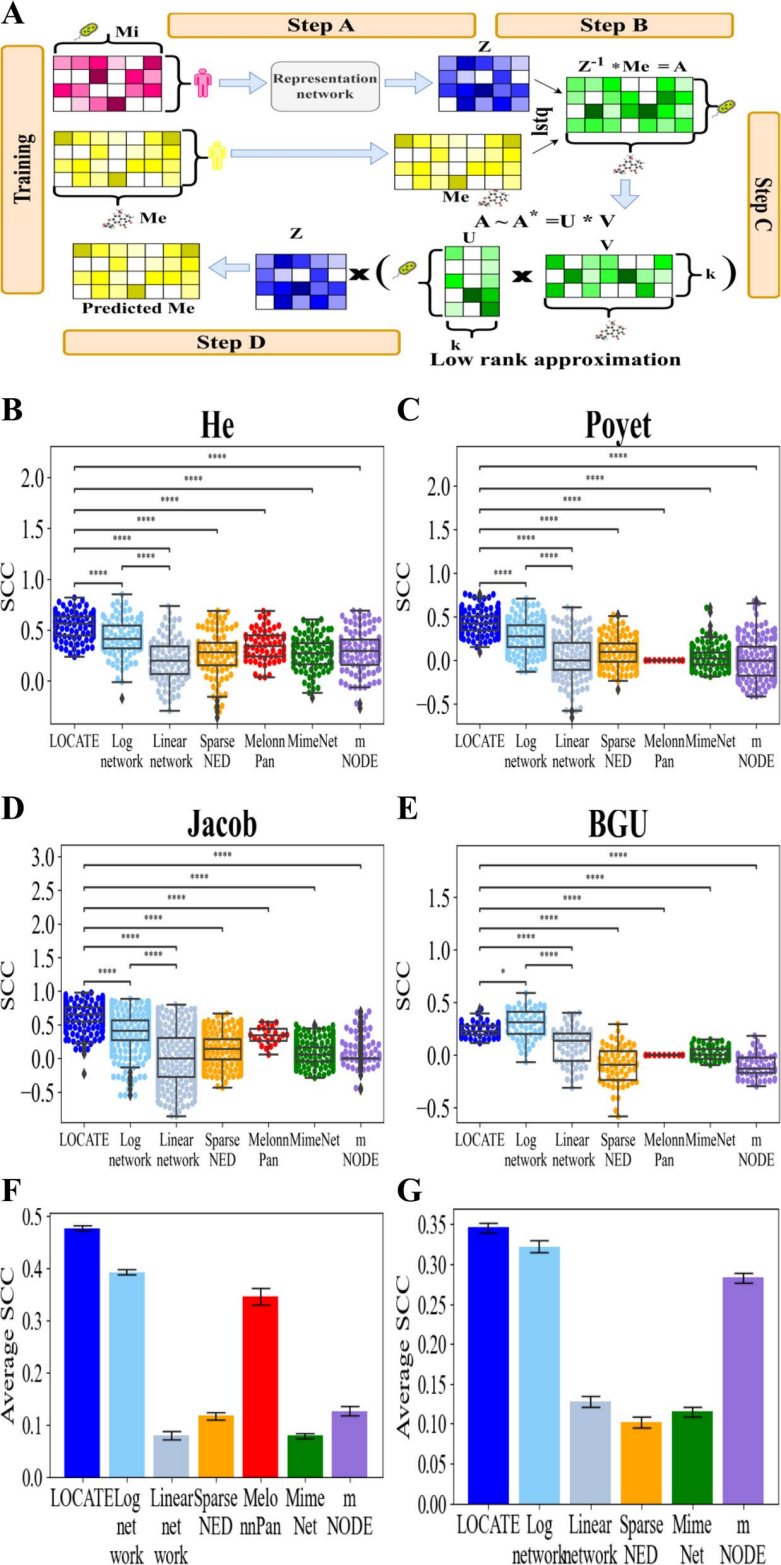
LOCATE can be used to predict metabolites in each dataset separately better than all existing methods. **A** A schematic figure of LOCATE’s training. Pairs of the preprocessed microbiome (*Mi*, in pink) and metabolites data (*Me*, in yellow) are the input of LOCATE. The preprocessed microbiome data is projected to a representation (*Z*) with a lower dimension than the microbiome using a fully connected neural network (step A). Then, *Z* is used to predict the metabolites of the training set. LOCATE finds a microbiome-metabolites relations matrix A, such that $A = z^{-1}*Me$ (step B). *A* is then passed through an SVD with low-rank approximation to prevent an overfit ($A^{*}$, step C) and then is multiplied by *Z* to get the predicted metabolites (step D). This entire process is trained at once. **B**–**E** Comparison between LOCATE and all state-of-the-art metabolites prediction models over the different 16S datasets He (**B**), Poyet (**C**), Jacob (**D**), and Direct Plus (**E**) for the swarm plots on the rest datasets (Supplementary material Fig. S1). Each point represents the SCC of a single metabolite in the dataset. In MelonnPan, there are fewer points since it predicts only the “well-predicted” metabolites as defined in the original paper [57]. Furthermore, when all the SCCs are 0, the model fails in the prediction of this dataset. A two-sided *t*-test was applied between the SCCs of the different models. LOCATE is significantly better with $p-value < 0.0001$. The stars represent the *p*-values, such that *$p-value \le 0.05$, **$p-value \le 0.01$, ***$p-value \le 0.001$, ****$p-value \le 0.0001$. **F**–**G** Average SCCs over all metabolites and all the datasets per model, the 16S averages (**F**) and the WGS averages (**G**). The black error bars represent the standard errors over all metabolites and all the datasets

Given the frequent contribution of a single yet varying microbe to each metabolite’s concentration, we tested whether a relation between the log of the microbe composition and the log of the metabolite concentration would produce a better prediction (further referred to as the “Log network” model). The Log network model assumes that a matrix *A* connects the logged microbiome frequencies *Mi* to the logged metabolites concentrations, *Me* (similar to Fig. 3A, step B). To find this matrix and avoid an over-fit, we apply a singular value decomposition (SVD) and a low-rank approximation (similar to Fig. 3A, step C) on its result (prediction results of this variant can be found at Supplementary material Fig. S6B). The resulting low-rank approximated matrix $A^{*}$ (Fig 3A, step C) is multiplied by the log microbe frequencies to produce the concentration of the metabolites (Fig. 3A, step D).

A representation of the log of each metabolite as a linear combination of the log of the microbial taxa frequency would imply a purely multiplicative relation between microbes and metabolites. While this produces a significantly (*p*-value $< 0.05$) more accurate prediction than the linear relation (for 16S Fig. 3B–F light blue vs. light gray and for WGS Fig. 3G and Supplementary material Fig. S1A–E), it is also a non-realistic assumption. In order to produce a more realistic model, we propose to translate the log microbiome into an intermediate representation through a neural network (latent variables approach) (Fig. 3A, step A), and then relate this representation to the log metabolome assuming a linear relation. We denote this model LOCATE—Latent variables Of miCrobiome And meTabolites rElations (see the “Methods” section for details). Formally, a latent representation of the microbiome (*Z*) is computed by a fully connected network (FCN) (Fig. 3A, step A). Then, a similar solution to the Log network model is applied to the intermediate representation of the microbiome (*Z*) to translate *Z* into the logged metabolites, (*Me*) (Fig. 3A, steps B–D). *Z* will then be further used to predict the host condition. Note that this entire model is trained at once. Thus, *Z* is inherently trained to represent the relation between the microbiome and the metabolome.

To evaluate LOCATE, we measured the SCC between the real and predicted metabolites over 5 different 16S rRNA gene sequence-based datasets with 11 phenotypes and 5 different WGS datasets with 5 phenotypes (see Table S2). We compared the results to existing state-of-the-art models, such as MelonnPan, MimeNet, SparseNED, and mNODE as well as to a Linear network and a Log network model. LOCATE significantly outperforms the state-of-the-art models (*p*-value $$< 0.001)$$ on each dataset separately (for 16S Fig. 3B–E and for WGS Supplementary material Fig. S1A–E) and on the average of all the datasets and all the metabolites (for 16S Fig. 3F and for WGS Fig. 3G). LOCATE also significantly outperforms the Linear and Log-log models (for 16S Fig. 3B–F blue and gray colors, for WGS Supplementary material Figs. S1A–E and 3G). To summarize, modeling the metabolites and microbiome relations via an intermediate latent representation is better than all the existing methods of interaction networks or direct predictions and combined learning.

### Microbiome-metabolite relations are dataset specific

**Fig. 4:**
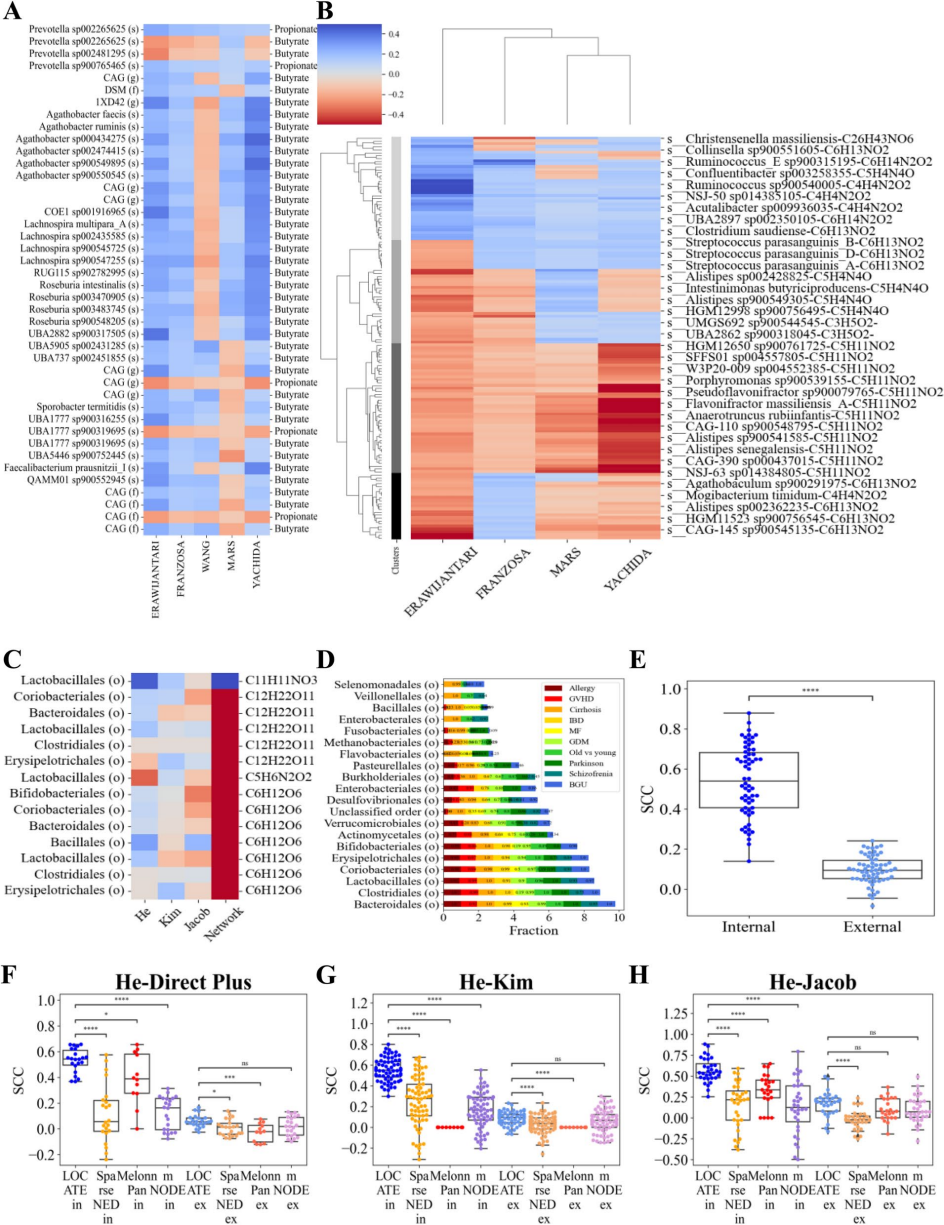
Microbiome-metabolite relations are dataset-specific. **A** Heatmap of significant SCCs between microbes and SCFA over different WGS datasets (ERAWIJANTARI, FRANZOSA, MARS, WANG, YACHIDA). Each row represents a microbe-metabolite pair and each column represents a different dataset. Red/blue colors represent negative/positive correlations. Many relations seem quite consistent. However, practically none of them is consistent over all datasets. **B** Heatmap of significant SCCs between all common microbes and metabolites over different gastric problems WGS datasets (ERAWIJANTARI, FRANZOSA, MARS, YACHIDA). Similar to **A**, each row represents a microbe-metabolite pair and each column represents a different dataset. **A** and **B** share the same color bar. The rows and columns are clustered. There are 4 different clusters of microbe-metabolite pairs. The first most light gray one consists of inconsistent pairs that tend to be positively related, the second darker gray cluster consists of equally inconsistent pairs, the third darker gray cluster consists of negatively correlated consistent pairs, and the last darkest cluster consists of inconsistent pairs that tend to be negative. The pair’s names in each cluster can be found in Supplementary material Table S5. **C** Heatmap of SCC between microbes and metabolites over different datasets (He, Kim, and Jacob) vs. the relations that are reported in the literature. The relations vary between different datasets and do not preserve the known relations from the literature. **D** The core microbiome. There are about 20 orders which are common to most of the datasets. These orders are also the most frequent taxa in the population of the cohorts. The *x*-axis represents the fraction of the population in the order that exists in each cohort. If the order appears in all the populations of all the cohorts, it sums to 10. The *y*-axis represents the different orders. Each color represents a cohort. **E** Swarm plot of LOCATE’s predicted metabolites SCCs in the cross-times test over the Direct Plus cohort. The dark blue points represent the SCCs of the prediction within a time point, referred to as “Internal,” where only one time point was used for the training and the testing, by the 10 CV approach. The light blue points represent the SCCs of the prediction between time points, where LOCATE is trained on one-time point (T0) and is tested on another one (T6). There is a decrease in the accuracy of the between-time points prediction. The stars follow the previous figure. For similar results on other time steps, see Supplementary material Fig. S4A–C. **F**–**H** Swarm plots of all of the cross-datasets predictions between couples of datasets on the shared metabolites and microbes, He-Direct Plus (**F**), He-Kim (**G**), He-Jacob (**H**); for similar results on the other pairs, see Supplementary material Fig. S4D–F. Each model is applied twice. First, it is trained on the intersection of the microbiome and metabolites of the pair but predicts on an internal test of the same dataset, “in-learning” (the dark points, referred to as “model-in”), then each model is trained on one dataset and is tested on the other dataset, “ex-learning” (the light points, referred to as “model-ex”). Training on one dataset and testing on another drastically decreases the performance of all the models, including LOCATE. However, LOCATE is still the significantly best model in most of the comparisons

Given the high accuracy of LOCATE, we used it to test whether the relation between microbiome and metabolome is conserved between conditions and datasets, or whether it is affected by the experimental procedure and host conditions. To test for dataset dependence, we first checked the association between metabolome and microbiome on the samples directly on the measured concentration. Given the importance of SCFA, we focused on those. The SCCs between the existing SCFA in the cohort and each microbe were calculated, and significant SCCs (*p*-value $< 0.05$) were considered. There are 141 different microbe-metabolites common pairs over the 5 WGS datasets. The microbial SCFA relations are indeed consistent over different datasets, with minor exceptions in several pairs especially in the WANG and MARS datasets (Fig. 4A). However, the consistency in microbes and metabolites relations is not universally conserved for all metabolites. Repeating the same computations for all metabolites over 4 WGS datasets of gastric problems ERAWIJANTARI, FRANZOSA, MARS, and YACHIDA, 4 types of pairs emerge (Fig. 4B). Most pairs are inconsistent among datasets, especially the positive correlations (the first bright gray cluster). Some are totally inconsistent (the second darker gray cluster). There are consistently negatively correlated pairs (the third darker gray cluster), and inconsistent pairs that tend to be negatively correlated (the last darkest cluster). Given the discrepancy in most pairs, we assume the microbe-metabolite relations are associated with external features. An even more extreme inconsistency can be seen when comparing the data sets of the 16S (Fig. 4C). Note that this analysis is applied at the order level of the microbiome to ensure a large enough intersect between the microbes present in different datasets (Supplementary material Fig. S2), which is extremely low (Supplementary material Figs. S2A and S3A–D). To further test the consistency between datasets, we analyzed each metabolite-microbe pair appearing in at least two datasets and computed the average SCC between each microbe-metabolite pair among all datasets containing the pair. The distribution of results is very narrow, around zero (−0.003 $+/- 0.08$ (Fig. S3I)), suggesting that there is practically no pair with consistent positive or negative correlation. Furthermore, when comparing the raw correlations with the relations that are reported in the literature [50], there are many contradictions (Fig. S3J). For an example of the inconsistent correlations across datasets and differing literature, see [50] (Fig. 4C). The same phenomenon appears when comparing the weights of the Log network coefficient matrix of different datasets (Supplementary material Fig. S3K). Note that the correlations are at the univariate level (a single microbe vs. a single metabolite), while the coefficients are the results of a multivariate analysis.

To further test for dataset dependence, we applied cross-dataset learning, where all models are trained on one cohort and are tested on another cohort, or a cross-condition prediction, where the models are trained on one condition in the dataset and tested on another in the same dataset. To ensure that the results are not induced by the technical details of a specific model, we repeated the analysis for multiple models. When applying the cross-datasets analysis, one may encounter a technical limitation. At the order level, most of the orders are unique to a specific dataset with 17% shared orders on average between 3 datasets (Supplementary material Fig. S3A–D). The intersection between datasets is even lower at finer taxonomy levels (Supplementary material Fig. S2A vs. B). The overlap between pairs is higher in the WGS pairs than in the 16S pairs, especially at the species level. Surprisingly, the intersection between the 16S and WGS is lower than the intersection within the 16S pairs. A quite similar situation happens in the metabolites. The average fraction of shared metabolites between the 3 datasets is 0.0114 (for the intersection of specific triads, see Supplementary material Fig. S3E–H). However, there is a core microbiome of about 20 orders which appears in high amounts in most of the datasets (Fig. 4D).

To apply cross-dataset learning, one could use the microbiome common orders, defined as the core microbiome (Fig. 4D), and predict only the shared metabolites. Two kinds of learning were evaluated. The first is referred to as “in”-learning and is based solely on the core microbiome in a given dataset. The second is referred to as “ex”-learning is applied to the core microbiome between datasets (i.e., only microbes present in high frequency in both datasets). In the “ex”-learning setup, the training is on one dataset and the testing on the other. The metabolite concentration prediction’s accuracy in the “in”-learning is similar to the prediction using the entire microbiome (Fig. 3 vs. Fig. 4F–H “in”). However, in the “ex”-learning, the metabolite concentration prediction’s accuracy over all the models is much lower (Fig. 4F–H “in” vs. “ex”).

The same decrease is observed in the Log network without the intermediate representation of LOCATE (Supplementary material Fig. S4G). Note that even in the cross-dataset predictions, LOCATE significantly outperforms the existing state-of-the-art models in most of the pairs both in the “in”-learning and the “ex”-learning (Fig. 4F–H, for other pairs Supplementary material Fig. S4D–F). The same decrease in accuracy is observed even in a given dataset with the same sequencing, the same machines, and the same cohort’s participants in different time points (T0 vs. T6) of the Direct Plus experiment (Fig. 4E, for other time points Supplementary material S4 A-C). The decrease in accuracy in the “ex”-learning of the cross-datasets analysis may result from a “context” dependent relation between the microbiome and metabolites. We propose to use this dependence to predict the host condition.

### Internal representation is associated with dataset features

**Fig. 5:**
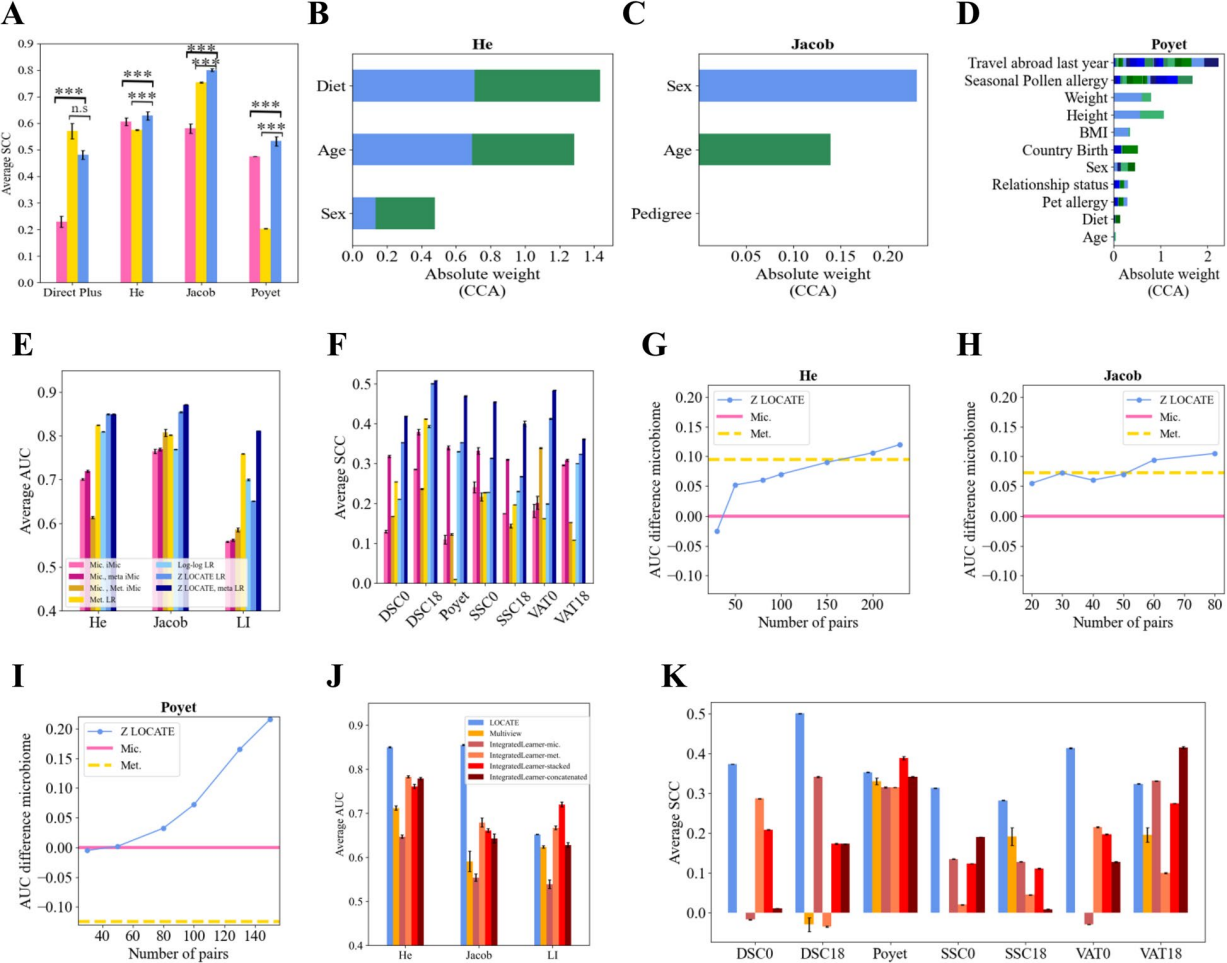
Internal representation improves outcome prediction compared with microbiome and metabolites and is associated with dataset features. **A** Average SCC between the CCA outputs of the microbiome and metadata (pink), the metabolites and metadata (yellow), and LOCATE’s representation and the metadata (blue). A one-sided *t*-test is applied between the models. The stars follow the previous figures. **B**–**D** Weights of the CCA between LOCATE’s representations and the metadata on its two first components on He (**B**), Jacob (**C**), and Poyet (**D**). When the variable is categorical, all the weights are stacked together in different colors (for the categorical information, see Supplementary material Table S7). The first component values are in blue colors and the second component values are in green. **E**, **F** Bar plots of average AUC (**E**) and the average SCC (**F**) of the predicted outcomes over different datasets and different tasks. The pink colors represent the different microbiome-based models. The light pink represents an iMic model trained on the microbiome data only (referred to as “Mic. iMic”). The dark pink represents an iMic model trained on the microbiome and the metadata together (referred to as “Mic., meta iMic”). The yellow colors represent the metabolites-based models. The light yellow represents a logistic regression (LR) model in **E** or a Ridge model in **F** trained only on the metabolites (referred to as “Met. LR”) and the dark yellow represents an iMic model trained on both the metabolites and microbiome (referred to as “Mic., Met. iMic”). The blue colors represent the models based on LOCATE. The lightest light blue represents the Log network (referred to as “Log-log LR”). The intermediate blue represents a model trained on LOCATE’s representation (referred to as “Z LOCATE LR”), while the darkest blue represents a model trained on both LOCATE’s representation and the metadata (referred to as “Z LOCATE, meta LR”). The standard errors between the 10 cross-validations are in black. A one-sided *t*-test was applied between the models. The *p*-values $< 0.001$ in all the comparisons apart from Kim and some of the LI tasks. **G**–**I** Effect of a decreasing number of metabolites for LOCATE’s representation on the condition predictions in He (**G**), Jacob (**H**), and Poyet (**I**). The *x*-axis represents the number of pairs of microbiome and metabolites used for the training of LOCATE; the *y*-axis represents the difference between the average AUC (over 10 runs) of the predicted outcome based on LOCATE’s representation and the average AUC (over 10 runs) of the predicted outcome based on the microbiome only. In most of the datasets, 50 metabolites are enough for LOCATE’s representation to be better than the microbiome. The pink line represents the zero value, and the dashed yellow line represents the metabolites’ contribution (of all samples) to the microbiome. When LOCATE is better the point is above the pink line. **J**, **K** Bar plots of average AUC (**J**) and the average SCC (**K**) of the predicted outcomes over different datasets and different tasks. The orange color represents the Multiview model’s results. The red colors represent the IntegratedLearner different models. The pink-red color represents an IntegratedLearner variant of microbiome only, the orange-red color represents an IntegratedLearner variant of metabolites only, the red color represents an IntegratedLearner variant of stacked, and the dark red color represents an IntegratedLearner variant of concatenated. The blue color represents LOCATE. The standard errors between the 10 cross-validations are in black. A one-sided *t*-test was applied between the models. The *p*-values $< 0.001$ in all the comparisons apart from the LI task, Poyet, and VAT18. All the results of **A**, **E**, **F**, **J**, and **K** are reported as an average of 10 runs on an external test set

**Fig. 6:**
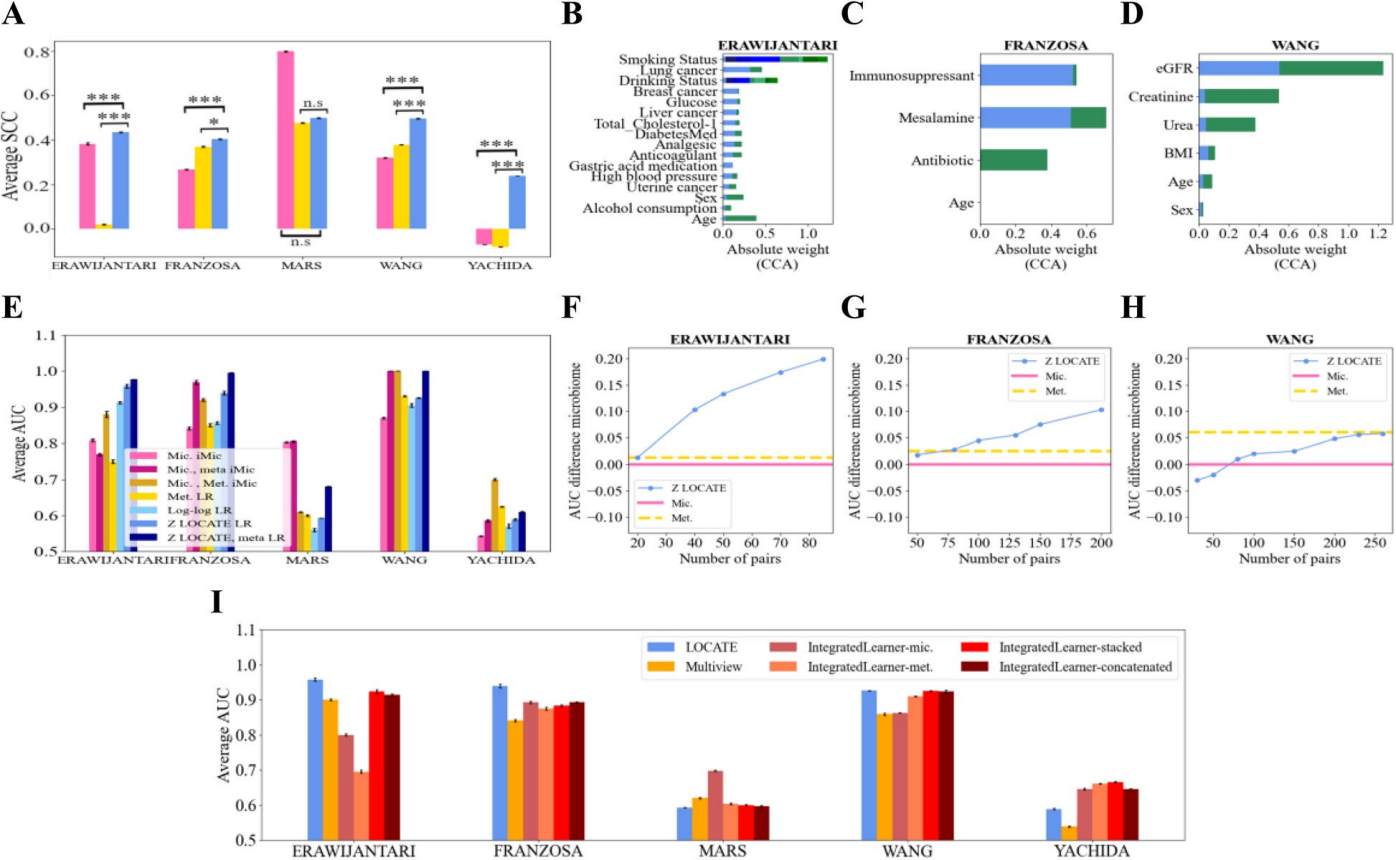
Internal representation improves outcome prediction compared with the microbiome and when possible also metabolites and is associated with datasets features—on WGS datasets. This figure follows the structure of the previous figure, but for WGS. The datasets used are ERAWIJANTARI, FRANZOSA, MARS, WANG, and YACHIDA for subplot **A** and for subplot **E**. In subplots **B**–**H**, we used ERAWIJANTARI, FRANZOSA, and the WANG datasets, with the same analysis in Fig. 5B–H. For the categorical variables of **B**–**D**, see Supplementary material Table S8. **I** This subplot follows Fig. 5J just on WGS datasets. The datasets used are ERAWIJANTARI, FRANZOSA, MARS, WANG, and YACHIDA

To test for a relation between the latent representation (*Z*) and demographic and health aspects of the hosts, such as their age, sex, etc., a canonical correlation analysis (CCA) was applied to relate either the microbiome or the metabolites or the latent representation to the available host characteristics. Then, the SCC was calculated between the first component of the CCA of each pair. The highest SCCs are obtained in the pairs of the representation vector and metadata with $p< 0.001$ in most of the comparisons (for 16S Fig. 5A and for WGS Fig. 6A). Further, the weights of the analysis of the first and second components are plotted. Quite consistently the weights of the age and sex are dominant (for 16S Fig. 5B–D and for WGS Fig. 6B–D).

### Internal representation improves host condition prediction compared with microbiome or metabolome separately or their combination

The stronger association of *Z* with demographics than either microbiome or metabolome by themselves may suggest that it can be a better predictor of host condition of interest in different experiments. To test for that, we compared the host condition prediction accuracy of different outcomes from binary conditions such as healthy vs. ill (e.g., IBD in the Jacob and FRANZOSA datasets, IBS in the MARS dataset, CRC in the ERAWIJANTARI and YACHIDA dataset, fatty liver, LI, in the Direct Plus dataset, and ERSD in the WANG dataset, infants diet (breast-feeding vs. formula) in the He dataset) to continuous conditions, such as age in the Poyet dataset and amounts of fats DSC, SSC, and VAT at different time points of the experiment in the Direct Plus dataset.

The prediction of cohort outcomes from LOCATE’s internal representation has a higher AUC/SCC for binary/continuous predictions than the microbiome-based predictions and most of the times also of the metabolites (Fig. 5E, F for 16S and Fig. 6E for WGS). For the microbiome-based predictions, we applied both logistic regression on the order level and iMic, which is the state-of-the-art in microbiome predictions on the species level. The LOCATE model is applied to both the microbiome of the order level and the species level, without significant differences between them. A logistic regression model is applied to LOCATE’s internal representations to predict the outcomes. The LOCATE’s representations are significantly more accurate (in most of the datasets and tasks apart from the Direct Plus, LI) with *p*-value $< 0.001$ better than using only the microbiome or only the metabolites as predictive features.

One may propose that *Z* is basically equivalent to demographic or additional data available in the different datasets, and as such is not useful beyond this demographic data. To test that this is not the case, we predicted the condition using a combination of *Z* and demographic data. The combination of *Z* and the additional data available for each data set (see Supplementary material Table S4) has higher accuracy than either by themselves or from the microbiome with the additional data (for 16S Fig. 5E, F and for WGS Fig. 6E). To summarize, we have shown that the latent representation *Z* is associated with the demographic properties of the host but adds more information to it about the condition than either the microbiome or metabolome.

Moreover, predicting the host condition based on the intermediate representation (that consists of the microbiome-metabolome interactions) is better (apart from LI in the 16S) than predicting the condition from the predicted metabolites of the Log network model (Fig. 5E, F) as well as from the combined microbiome and metabolites model (apart from YACHIDA in the WGS, Fig. 6E).

Often, given the cost difference between metabolome profiling and microbiome sequencing, one aims experimentally to measure the metabolite concentrations on a sub-group of the samples. Given the fact that once the model is trained, *Z* is only computed using the microbiome, one may propose to measure metabolites and train the model on a partial set of samples, and then compute *Z* for all the microbiome samples. To test for such a hybrid sampling method, we computed the minimal number of samples required for training LOCATE’s representation, such that the prediction accuracy would be higher than the one of the microbiome on the test set. For most datasets, using 50 metabolite samples and above for the training is enough to improve the overall accuracy (for 16S Fig. 5G–I and for WGS Fig. 6F–H). Note that the improvement in condition prediction presumes a relation between the metabolites and the condition. Therefore, it is recommended to first check for this relation through the metabolite reconstruction accuracy, and only then to apply LOCATE to predict a condition.

To get a more holistic comparison, we also compared LOCATE’s results to other multi-omics approaches, such as Multiview and IntegratedLearner both on 16S cohorts and WGS cohorts. LOCATE significantly outperforms the state-of-the-art multiview approaches (for 16S Fig. 5J, K and for WGS Fig. 6I).

## Discussion

Two different tasks are often performed when combining microbiome and metabolome studies. The first is to predict the metabolome from the microbiome (the opposite is rarely done to the best of our knowledge, apart from a single work of predicting the gut microbiome alpha diversity from the metabolites compositions [79]), and the second is to combine both metabolome and microbiome to predict a phenotype of the host or any other property of each host [25]. The first was typically done by a linear or non-linear translation of the microbiome into some or all of the metabolites (sometimes only a subset of the metabolites in the sample are predicted), assuming that the microbiome determines the metabolite concentration. The second task is typically performed by combining the two types of data and performing a prediction of the target condition.

Here we propose that the first task should be treated through the creation of a latent representation (*Z*) of the microbiome and metabolome, using LOCATE. This representation is host and condition-specific. A similar concept, with a different mathematical approach, has been previously proposed [71, 80]. This representation is associated with the sample context which can be the age, gender, dietary habits, or health condition of the host. We then show that *Z* is strongly associated with the host demographic, diet, and other features. Finally, we show that it better predicts the host properties than either the microbiome or metabolome. As such it serves to combine the two tasks above. The main difference between this approach and most existing combinations of microbiome and metabolome to predict condition [25, 58, 59] is that instead of combining the two, we propose to find intermediate variables between the two and use those to predict the condition. We denote this representation *Z* all along and the algorithm producing it LOCATE.

By combining the solution on the above two tasks, LOCATE is less sensitive to the limitations of condition prediction by either microbiome or metabolome. It is more directly associated with host properties as measured by a CCA to measure host properties than either microbiome or metabolome. A crucial aspect of LOCATE is that it provides a low dimensional representation of the host (10 dimensions in the current analysis). Such a low dimension makes the representation amenable to easy manipulation with no need for detailed knowledge of either microbes or metabolomes (for untargeted metabolites contribution to the precision of condition prediction from microbiome-metabolome combinations compared to the prediction based only on classified metabolites, see Supplementary material Fig. S7).

At the practical level, we show that it is enough even for experiments with a large number of samples to measure less than 100 metabolome samples. Those can then be integrated with the microbiome using LOCATE, and the internal representation of all other samples can be computed from the microbiome via LOCATE. As such, it can serve as a viable solution for large cohorts at a reasonable cost. This solution is applicable to both 16S and WGS.

The obvious caveat of LOCATE is that it does not provide a measure that can be directly compared between experiments. As such, its use is limited (as are all current microbiome-metabolome combinations) to a given experimental setup. The development of a cross-platform latent variable host representation that could merge 16S and WGS would require domain-invariant machine learning algorithms. A classical solution would be the combination of LOCATE with an adversary classifier that should fail to distinguish between experiment [81, 82]. Such a system could in theory distill the representation relevant to the host biology from the ones related to the experimental procedure. We have tested such possible systems. However, they seem to require more and larger experiments than are currently available.

Beyond its application as a prediction tool for metabolite and host conditions, LOCATE can also be used to define a distance between samples since it provides an internal low-dimension latent representation. Such a distance can be used among many others for visualization through a PCoA projection to 2 or 3 dimensions. It can be used for sample clustering and anomaly detection. The analysis of the statistical properties of this distance would require more datasets than currently used and is left to further studies.

LOCATE is available as a GIT at https://github.com/oshritshtossel/LOCATE and as a PyPI at https://pypi.org/project/LOCATE-model/. All the datasets used are from published cohorts, see Supplementary material Table S2. All the raw microbiome and metabolome data are available as supplementary tables, apart from the tables of the Direct Plus dataset. This dataset belongs to Prof. Iris Shai and can be acquired by direct contact with this group.

## Conclusions

The association of microbes with metabolites is treated oversimplistically as a direct relation of microbes consuming a metabolite increasing in its presence, and similarly the concentration of metabolites produced by a microbe increasing in its presence. We have shown that a much more complex relation should be considered, where the metabolites and microbes produce a condition-dependent equilibrium. On the one hand, one cannot simply predict the change in a microbe through a change of the metabolites. On the other hand, this equilibrium allows for a precise prediction of the condition based on the combined metabolite and microbes of a set of samples. Interestingly, even with a small number of metabolites, a latent representation of the environment can be produced from the relation between microbes and metabolites. This representation can then be used with large microbiome samples to predict with high accuracy disease states or other conditions associated with the gut-metabolome.

## Methods

In order to facilitate the understanding of the more mathematical and ML-oriented terms in the text, we provide a short description of the main ML terms used in the manuscript in Supplementary material.

### Datasets

We analyzed data from multiple published studies of the human gut microbiome and metabolome. We focused on studies that included at least 90 individuals, following the rules proposed by Borenstein’s gut microbiome-metabolome dataset collection [80], for which both the microbiome and the metabolome were profiled from fecal samples. We used five 16S rRNA gene sequencing paired datasets and five whole genome shotgun sequencing (WGS) paired datasets. For more details on each cohort, see Supplementary material Table S2.

#### 16S rRNA gene sequencing-based paired datasets

##### Direct Plus

Longitudinal samples of fecal microbiome and metabolites (over 18 months) of 294 participants with abdominal obesity/dyslipidemia into healthy dietary guidelines (HDG), MED, and green-MED weight-loss diet groups, all accompanied by physical activity [83]. The outcomes we studied here were deep subcutaneous (DSC), superficial subcutaneous (SSC), visceral adipose tissue (VAT), and fatty liver. During this analysis, we used only the microbiome and metabolites from the first time point (T0) and the last time point (T18) separately for each participant.

##### He

Microbiome and metabolites from infants over several time points during the first year of life, either breastfed, formula-fed, or experimental formula fed. [84]

##### Jacob

IBD patients, 21 Crohn’s disease (CD) and ulcerative colitis (UC) probands younger than the age of 18 were recruited from the Pediatric IBD Center at the Cedars-Sinai Medical Center and their first-degree relatives of patients with IBD. We analyzed both their microbiome and their metabolites of them [85].

##### Kim

Fecal microbiome and metabolites of patients with advanced colorectal adenomas, colorectal cancer, and controls [86].

##### Poyet

Longitudinal samples from healthy donors to the Broad Institute-OpenBiome Microbiome Library (BIO-ML) [87].

#### WGS paired datasets

##### ERAWIJANTARI GASTRIC CANCER 2020

Fecal and metabolites of patients who underwent colonoscopy, half of whom with a history of gastrectomy for gastric cancer and no signs of gastric cancer recurrence [88]. This dataset is referred to as ERAWIJANTARI.

##### FRANZOSA IBD 2019

Fecal microbiome and metabolites of IBD patients and controls (PRISM cohort + A validation cohort) [89]. This dataset is referred to as FRANZOSA.

##### MARS IBS 2020

Longitudinal samples fecal microbiome and metabolites (over 6 months) from patients with irritable bowel syndrome (IBS) and controls [90]. This dataset is referred to as MARS.

##### WANG ESRD 2020

Fecal microbiome and metabolites of adults with end-stage renal disease (ESRD) and controls [91]. This dataset is referred to as WANG.

##### YACHIDA CRC 2019

Fecal microbiome and metabolites of patients who underwent colonoscopy, with findings from normal to stage 4 colorectal cancer [92]. This dataset is referred to as YACHIDA.

#### Microbiome preprocessing

For LOCATE, microbial data was pre-processed using the MIPMLP pipeline [93]. We merged the ASVs either to the order (to gain maximal intersection between pairs Supplementary material Fig. S2) or to the species taxonomic level by the Sub-PCA method (detailed below), but similar results are obtained at the other taxonomy levels as well (data not shown). Then, we applied log normalization (detailed below) on the merged ASVs. We further normalized each taxon such that its average will be 0 and its variance will be 1 (*z*-score). Notably, we examined variations of the LOCATE model with no *z*-scoring for the microbial data, yet for clarity, we present outcomes from the highest accuracy variant (additional variant results are available in Supplementary material Fig. S6).

For the other algorithms (SparseNED, MelonnPan, mNODE, and MiMeNet), we followed the preprocessing that was reported in the relevant papers [27, 57, 60, 61]. For the pre-analyses of Fig. 2, the ASVs were merged to the order level by the mean method (detailed below), and a relative normalization (detailed below) was applied (to keep the values positive).

Sub-PCA merging in MIPMLP: A taxonomic level (e.g., species) is set. All the ASVs consistent with this taxonomy are grouped. A PCA is performed on this group. The components which explain more than half of the variance are added to the new input table. This merging was applied for LOCATE.

Mean merging in MIPMLP: A level of taxonomy (e.g., species) is set. All the ASVS consistent with this taxonomy are grouped by averaging them. This merging was applied to the NMF and to the analyses of Fig. 2.

Relative normalization in MIPMLP: To normalize each taxon through its relative frequency

1 $$\begin{aligned} x_{i,j} = \frac{x_{i,j}}{ \sum _{k=1}^{n} x_{k,j}}, \end{aligned}$$

we normalized the relative abundance of each taxon *j* in sample *i* by its relative abundance across all *n* samples. This was applied only to the NMF model and to the analyses of Fig. 2.

Log normalization in MIPMLP: We logged (10 base) c-wise, according to the following formula:

2 $$\begin{aligned} x_{i,j} \rightarrow \log (x_{i,j}+\epsilon ), \end{aligned}$$

where $\epsilon$ is a minimal value $(= 0.1)$ to prevent log of zero values. This was applied for LOCATE.

#### Metabolite preprocessing

For LOCATE, all metabolic samples were first normalized to relative frequencies, such that the metabolites of each sample would sum to 1. Then, those were log-normalized and further *z*-scored, such that the average value of each metabolite would be 0 and its variance would be 1. Again for the other algorithms, we followed the preprocessing that was reported in the relevant papers [27, 57, 60, 61]. For the analyses of Fig. 2, only relative normalization was applied.

### Matrix factorization methods

We used a *Non-Negative Matrix Factorization (NMF) *that finds two non-negative matrices (W, H) whose product approximates the non-negative matrix *Me*. This factorization can be used for example for dimensional reduction, source separation, or topic extraction. In our case, we assumed $Me_{train}$ is the non-negative metabolites matrix of the training, and we express it as a product of the training microbiome matrix ($Mi_{train}$) and another relations matrix (*A*). Then, we used the training relations matrix to predict the metabolite values from the microbial abundances. We used the NMF decomposition of sklearn version 0.24 [94] with its default parameters apart from the L1 regularization that was fine-tuned and set to 10. The initialization matrix was initialized randomly with numbers between 0 to 1. We checked different initializations and they did not affect the convergence of the algorithm.

### Metabolites prediction by Latent variables Of miCrobiome And meTabolites rElations (LOCATE)

To predict the log normalized metabolite concentrations (*Me*, yellow in Fig. 3A) from the log normalized microbiome (*Mi*, pink in Fig. 3A), we first built an intermediate latent representation between the microbiome and the metabolites by using a fully connected neural network (*Z*, blue in Fig. 3A, step A). Representation network: A 3-layer fully connected neural network FCN was applied to the log-normalized microbiome data (different dimension reduction methods, such as 1D-CNN and deep networks, were also tested, Supplementary material Fig. S6C and D). An activation function (either of RelU, elU, or Tanh) was applied between the layers, and dropout and L2 regularization were also applied. The representation dimension was set to 10. All the network hyperparameters, except for the representation dimension, were chosen via the Neural Network Intelligence (NNI) platform [95] on each dataset separately on an internal validation set. The hyperparameters used can be found in Supplementary material Table S3. The loss was a standard MSE loss,$\frac{1}{N}\sum _{i=1}^{N}\sum (Me_i - \hat{Me_i})^2$, where *N* is the number of the microbiome and metabolites paired samples and $\hat{Me_i}$ is the predicted metabolites, and an Adam optimizer was used (Fig. 3A, step A).

The output of the neural network was the input of a linear predictor of the log metabolite concentration. We assumed a microbiome-metabolites relationship matrix *A* (green in Fig. 3A) such that

3 $$\begin{aligned} A\cdot Z = Me \rightarrow A=Z^{-1}\cdot Me_{train}, \end{aligned}$$

where $Z^{-1}$ is the pseudo inverse of *Z* ($(Z^t\cdot Z)^{-1}\cdot Z^t$), since *Z* does not have to be a squared matrix. The pseudo-inverse was computed using the *torch.linalg.lstsq* function on *Z* and $Me_{train}$ (Fig. 3 step B). To prevent overfitting, we did not use *A* directly but applied a low-rank approximation on *A* using *torch.svd_lowrank* with its default parameters (Fig. 3 step C). In the inference step, the low-rank approximated matrix of $A^*$ (an approximating matrix with reduced rank) from the training was used (Fig. 3 step D). It is important to note that the neural network’s end-to-end training produces a representation (*Z*) that encodes the combined information of both microbiome and metabolites (via the backpropagation), even though its direct connection to metabolites might not be as explicit as that of A. We also tested LOCATE without the low-rank approximation (Supplementary material Fig. S6 B).

### Host condition prediction

To test which variables best explain the condition of the cohorts, we predicted the condition once from the original microbiome (*Mi*) at two different taxonomy levels, the order level, and the species level, once from the original metabolites (*Me*) and once from LOCATE’s latent microbiome-metabolite representation (*Z*). For binary conditions, a logistic regression model was applied with its default parameters, including an L2 regularization of 1, of the sklearn library. For continuous conditions, a Ridge regression model was applied with its default parameters of the sklearn library. Note that no hyperparameter tuning was applied. The data was split into a training set (80% of the data) and a test set (20% of the data), and we reported the results on the test set as an average of 10 different splits as described in the “Statistics and evaluation” section. For the microbiome-based learning at the species taxonomic level, the microbiome was translated into a 2D image, such that each row of the image represents another taxonomy level according to the cladogram structure. Then, a novel CNN-based prediction, iMic[31], was applied for both the regression and classification models. For the robustness of condition prediction models against overfitting, see Supplementary material Fig. S5.

### Statistics and evaluation

#### Spearman correlation coefficient (SCC) and area under the receiver operating characteristic (ROC) curve (AUC)

To evaluate the prediction quality of the metabolite predictions, we calculated the Spearman correlation coefficient (SCC) between the real metabolites and the predicted metabolites over two different frameworks: *within a given dataset*—by removing 20% of the data for testing, and *cross-datasets approach*, training the model on one dataset and testing it on another.

We further used the SCC to evaluate the condition prediction of the continuous conditions by measuring the SCC between the predicted phenotype and the real phenotype on the test set. An average of 10 cross-validations on the test set was reported.

To evaluate the condition predictions of the binary phenotypes, the AUC of each model (microbiome-based, metabolites-based, and representation-based) was calculated on a test set. An average of 10 cross-validations was reported on the test.

#### Representation matrix and metadata relationships

To test the relations between the demographic features (e.g., age, gender, etc.) of each cohort and its representation (*Z*), we first applied canonical-correlation analysis (CCA) [96] between the original microbiome (*Mi*) input and the metadata, the original metabolites (*Me*) data and the metadata and the representation (*Z*). Then, we trained 10 CCA models on each of the training sets (80% of the data each) and predicted the CCA for each of the 10 test sets (20% of the data each) separately. Then, the average SCCs (over the 10 models’ partitions) between the real CCAs and the predicted ones were computed and reported with their standard errors over the 10 runs. We further predicted the metadata once from the microbiome (*Mi*), once from the metabolites (*Me*), and once from the representation (*Z*) using a Ridge model. The average SCC between the predicted metadata and real metadata was computed on the test set. To detect the metadata features that are related to the microbiome-metabolites representation (*Z*), the absolute CCA’s weights of the two first components were computed.

### Experimental design

#### Generate a uniform platform for metabolites

Each dataset had a different notation for the metabolites. Consequently, we translated the identity of each metabolite to its chemical formula by using the API of the following websites Metabolomicsworkbench, and KEGG COMPOUND Database [49, 97] as well as by the PubChemPy python package https://github.com/mcs07/PubChemPy.

#### Training and test sets split

##### Representation learning

Within dataset: We divided the data with an external test of 20% of the whole data. The remaining 80% of the data was used as the training set. We repeated the split 10 times, such that the reported results were an average of the 10 runs.

Cross-datasets prediction: First, we merged the dataset by removing microbes and metabolites that did not appear in the intersect of the datasets. Then, each dataset was normalized separately. Next, two different types of learning were applied: (1) *“in”-learning*—given one single dataset with only its core microbiome and shared metabolites, applying learning within the dataset by dividing it into a training set (80% of the data), and a testing set (20% of the data). (2) *“ex”-learning*—where one dataset was used for training, while the other dataset was used for testing.

Condition predictions: The data (microbiome and condition, or metabolites and condition, or LOCATE’s representation, *Z*, and condition) was divided into 2 groups; 80% of the data was used for training and the remaining 20% was used for testing. We repeated the split 10 times, such that the reported results were an average of the 10 runs.

#### Creating the representation on a varying number of training samples

We aimed to determine the optimal sample size for pairs of microbiome and metabolite data that would yield a robust microbiome-metabolite representation through LOCATE, thus enhancing condition prediction accuracy. To this end, we conducted an investigation involving varying numbers of microbiome-metabolome pairs (for example ranging from 25 to 225 in the He dataset), mirroring the common scenario where there are fewer metabolite data samples than microbiome samples. This diverse sample range was chosen to reflect the typical scope of experiments conducted. Our approach involved training the representation network of LOCATE, using the specified number of paired samples. Subsequently, we inferred representations for all samples within the cohort, even those lacking metabolite data, leveraging the trained model.

### Supplementary information

**Additional file 1. **

## Data Availability

All the datasets used in this manuscript are from published papers, as can be seen in Table S2. The WGS datasets were downloaded from [80]. The raw pairs of the microbiome and metabolites datasets (apart from the Direct Plus cohort) are also uploaded as Supplementary material. Overall analysis code including statistical analysis, comparisons, and visualizations is available at https://github.com/oshritshtossel/LOCATE_all_analysis. LOCATE model is available as a PyPi https://pypi.org/project/LOCATE-model/ and as a separate GitHub https://github.com/oshritshtossel/LOCATE.
