# Supplementary material for: Gut microbiome-metabolome interactions predict host condition
Source: Microbiome. 2024 Feb 10;12:24. doi: 10.1186/s40168-023-01737-1 (PMC10858481; doi:10.1186/s40168-023-01737-1)
Supplement: Supplementary file 2 — Additional file 1. [file 40168_2023_1737_MOESM1_ESM.pdf]

Supplemetary Material

September 13, 2023

1 ML nomenclature

In order to facilitate the understanding of the more mathematical and Machine Learning (ML) oriented terms in the text, we provide a short description of the main ML terms used in the manuscript.

- **Model** is the mathematical relation between any input (in our case, microbiome ASVs, or metabolites or LOCATE's representation, Z) and the appropriate output (in our case the class of the sample/the phenotype). In ML, the model usually contains a set of parameters called weights, and the ML trains the model by finding the weights for which the model is in best agreement with the relation between the input and output in the "Training set".
- **Training set** The part of the data used to train the model. The quality of the fit between the input and output data on the training set is not a good measure of the quality of the model, since it may be an "overfit".
- **Overfitting** A problem occurs when a model produces good results on data in the training set (usually due to too many parameters), but produces poor results on unseen data.
- **Validation set** is a separate set from the training set that is used to monitor but is not used for the training process. This set can be used to optimize some parts of the learning process including setting the "hyperparameters".
- **Model hyperparameters** are adjustable values that are not considered part of the model itself in that they are not updated during training, but still have an impact on the training of the model and its performance. To ensure that those are not fitted to maximize the test set performances, the hyperparameters are optimized using an internal validation set.
- **Test set** Data used to test the model that is not used for either hyperparameter optimization or the training. The quality estimated on the test set is the most accurate estimate of the accuracy.
- **k-Fold Cross-Validation (referred to as k CVs)** is a resampling procedure used to evaluate machine learning models on a limited data sample. The data is first partitioned into k equally (or nearly equally) sized segments or folds. Subsequently, k iterations of training and validation are performed such that within each iteration a different fold of the data is held out for validation while the remaining k-1 folds are used for training.
- **Receiver Operating Characteristic Curve (ROC)** is a graph showing the performance of a classification model at all classification thresholds. This curve plots two parameters: True Positive Rate (TPR = is the probability that an actual positive will test positive); False Positive Rate (FPR = the probability that an actual negative will test positive).

- **Area under the ROC curve (AUC)** is a single scalar value that measures the overall performance of a binary classifier. The AUC value is within the range [0.5–1.0], where the minimum value represents the performance of a random classifier and the maximum value corresponds to a perfect classifier (e.g., with a classification error rate equivalent to zero). It measures the area under the ROC curve defined above.
- **Factorization** is the process of decomposing a matrix into the product of other smaller matrices.
- **Unit vectors** Vectors with a norm of one.
- **Orthonormal** Two vectors in an inner product space are orthonormal if they are orthogonal (or perpendicular along a line, meaning their inner product is zero), and have a norm of 1.
- **Singular Value Decomposition (SVD)** is the factorization of a matrix A (in our case, the microbiome-metabolite relation matrix) into the product of three matrices U, D and V^t, where the columns of U and V are "orthonormal" and the matrix D is diagonal with positive real entries. By SVD, one can determine the "matrix's rank", quantify a linear system's sensitivity to numerical error, or obtain an optimal "low rank approximation" to the matrix.
- **Low rank approximation** A simplified representation of a matrix obtained by retaining only the most significant components or factors, typically achieved through techniques like Singular Value Decomposition (SVD). Lower-rank approximations can reduce data dimensionality while preserving key information. This process helps improve the generalization ability of models or analyses, making it easier to identify and understand key biological relationships or features.
- **Latent representation** is the representation of a high-dimension vector by a lower dimension with the appropriate model keeping most of the information.
- **CCA** is a statistical technique used to explore and quantify the relationships between two sets of variables. In simpler terms, CCA helps us understand if there are meaningful connections between two sets of data (in our case, a view (microbiome/metabolites/Z) and host features.

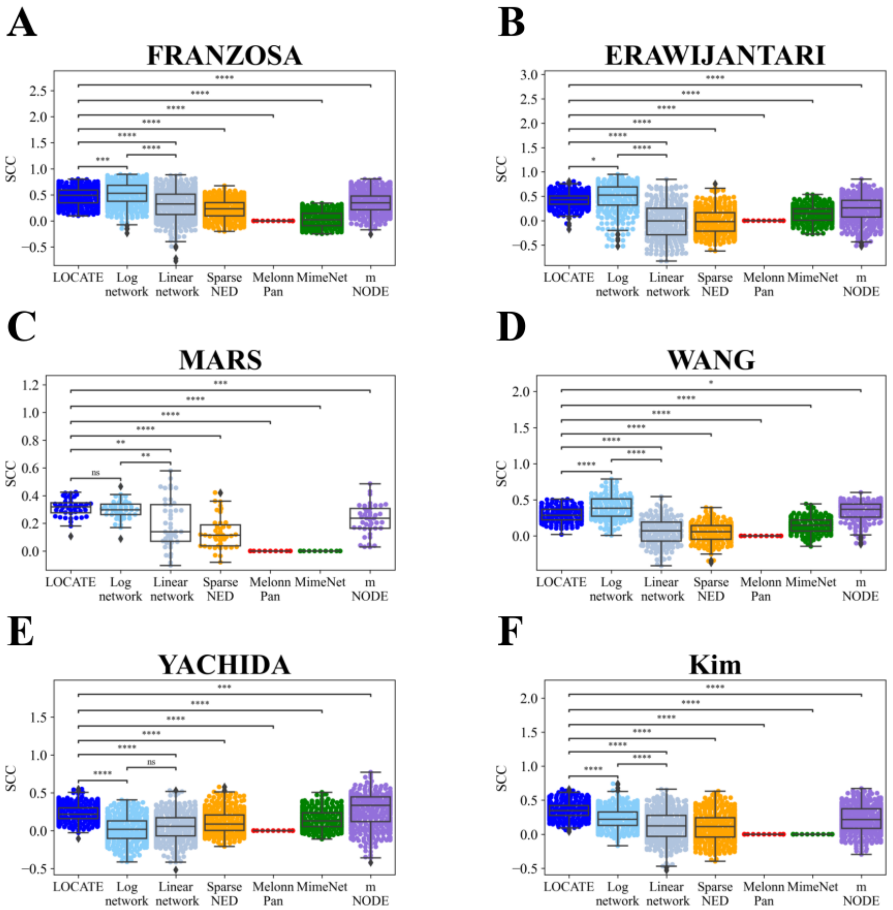

Figure 1: LOCATE can be used to predict metabolites in each dataset separately better than all existing methods. **A - E.** Comparison between LOCATE and all state-of-the-art metabolites prediction models as well as a Linear network and a Log network over the different datasets FRANZOSA **(A)**, ERAWIJANTARI**(B)**, MARS **(C)**, WANG **(D)** and YACHIDA **(E)**. **F.** Comparison between LOCATE and all state-of-the-art metabolites prediction models as well as a Linear network and a Log network over the Kim dataset.

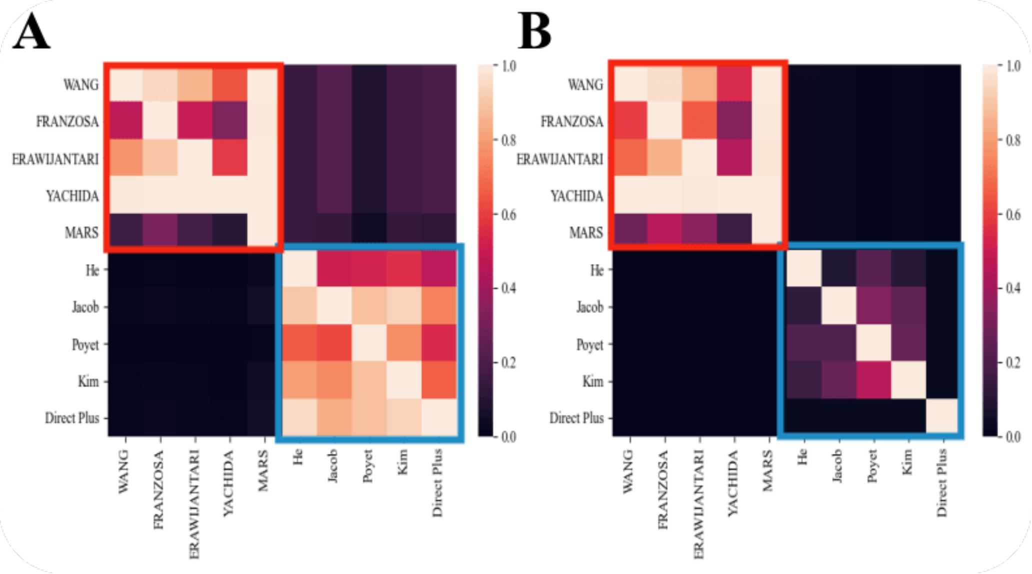

Figure 2: Intersections between pairs of cohorts of 16S and WGS at the order taxonomic level **(A)**, and at the species level **(B)**. The overlap between the pairs of the WGS datasets (red) is much higher than the overlap in the 16S datasets (blue), especially at the species level. The overlap between 16S and 16S is higher than the overlap between 16S and WGS, although the number of taxa in WGS is much higher than 16S, and one could expect the 16S taxa to be included in the WGS.

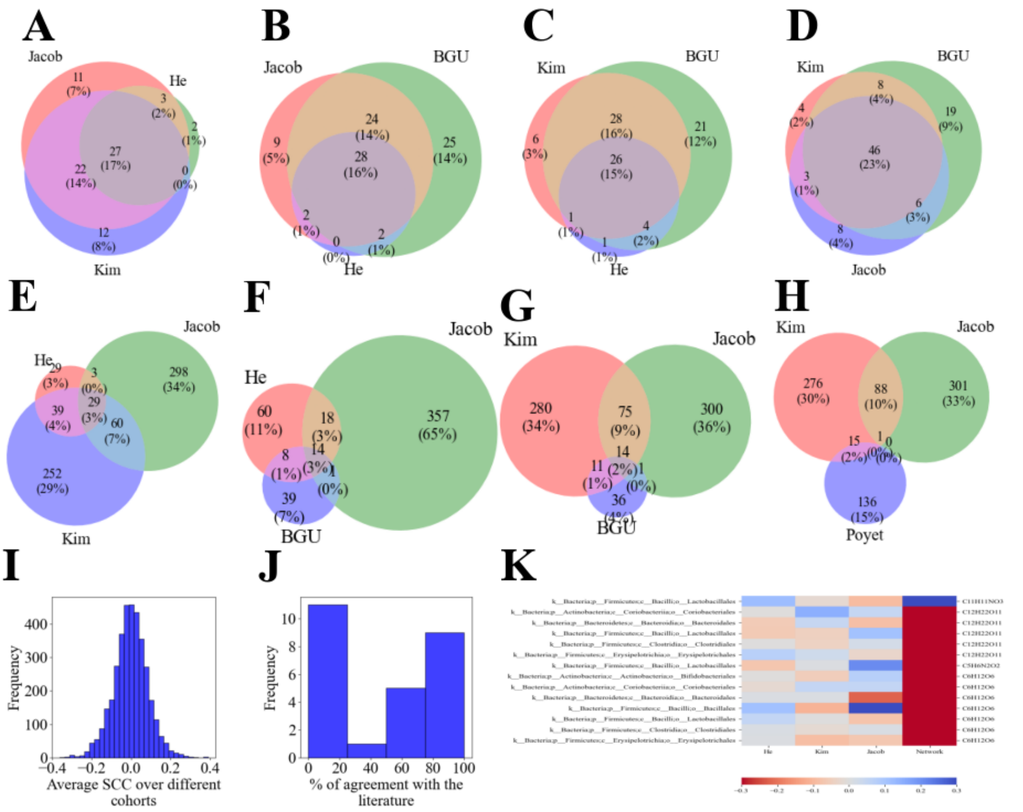

Figure 3: Low intersection between the orders microbiome and metabolites of different cohorts. **A - D.** Venn diagrams of the microbiome of triads 16S datasets. **E - H.** Venn diagrams of the metabolites of triads 16S datasets. Each color represents a dataset, and the intermediate colors represent the intersection. **I.** Histogram of average SCCs between each microbe and each metabolite that appears at least at 2 cohorts (of the 16S cohorts). The histogram's peak is at 0.0, which emphasizes the inconsistent SCCs cross datasets. **J.** Histogram of percent of agreement with the correlations reported in the literature and the correlations found in the cohorts. Most of the correlations do not agree with the literature. **K.** Heatmap of NMF coefficients between microbes and metabolites over different datasets (He, Kim and Jacob) vs the relations that are reported in the literature. Blue/Red colors represent positive/negative correlations. The relations vary between different datasets and do not preserve the known relations from the literature [1].

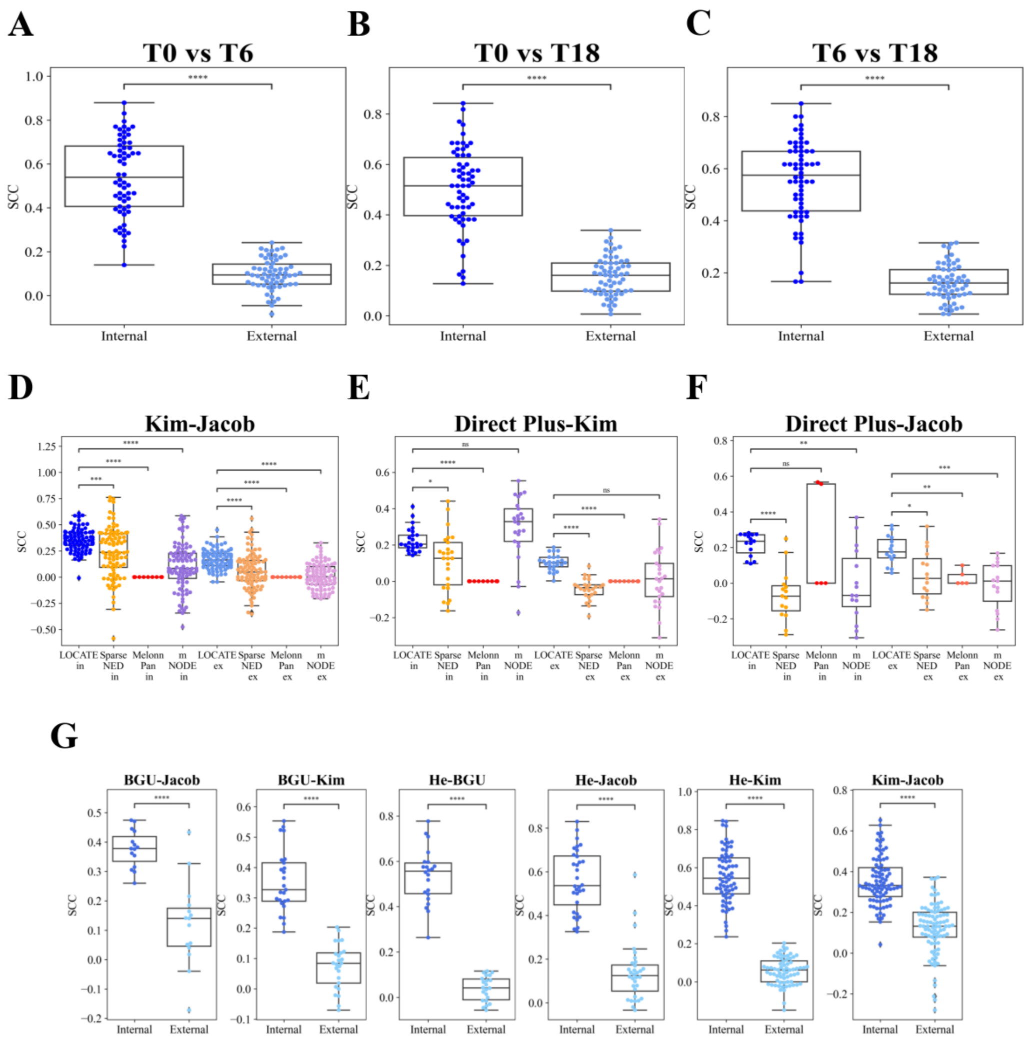

Figure 4: Microbiome-metabolite relations are dataset specific. **A - C.** Swarm plots of LOCATE's predicted metabolites SCCs in the cross-times test over the Direct Plus cohort. The dark blue points represent the SCCs of the "in-learning", referred to as "Internal", where only one time point was used for the training and the testing, by the 10 CV approach. The light blue points represent the SCCs of the "ex-learning", referred as "External", where LOCATE is trained on one time point and is tested on another one. There is a decrease in the accuracy of the ex-learning vs the in-learning. The stars follow all other figures. **D - F.** Swarm plots of all of the cross-datasets learning between couples of datasets, Kim-Jacob **(D)**, Direct Plus-Kim**(E)**, Direct Plus-Jacob **(F)**. **G.** Swarm plots of all of the cross-datasets learning between couples of datasets of the Log network model. The decline in performance between the "in-learning" and "ex-learning" can be seen here, too.

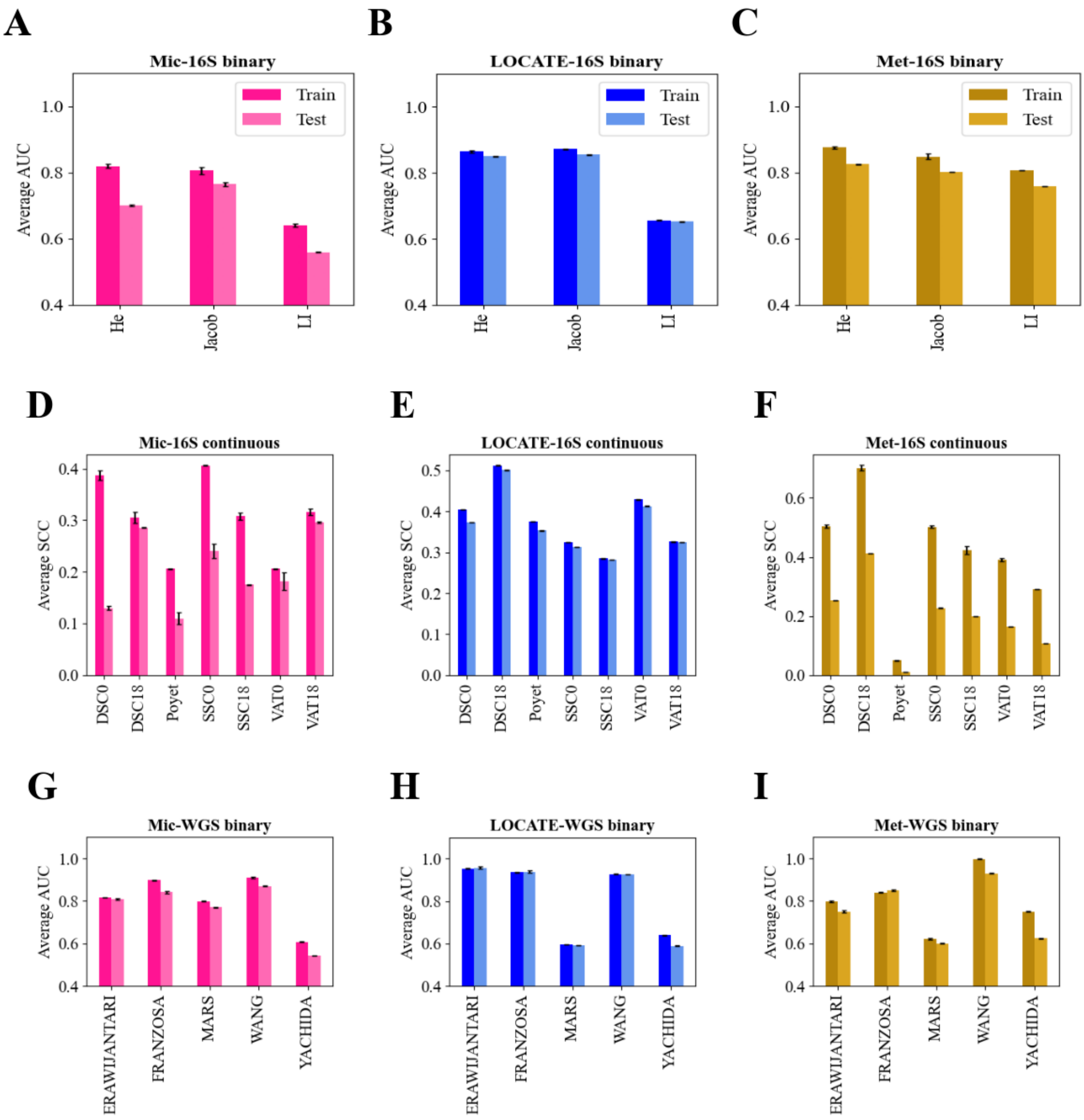

Figure 5: Robustness of host condition prediction models against overfitting. **A - C.** AUC comparison between training and test sets for binary tasks involving 16S cohorts in microbiome-based models (**A**), LOCATE models (**B**), and metabolite-based models (**C**). **D - F.** SCC comparison between training and test sets for continuous tasks involving 16S cohorts in microbiome-based models (**D**), LOCATE models (**E**), and metabolite-based models (**F**). **G - I.** AUC comparison between training and test sets for binary tasks involving WGS cohorts in microbiome-based models (**G**), LOCATE models (**H**), and metabolite-based models (**I**). Dark bars denote training performance, while light bars signify test set performance. The black error bars represent the standard errors within the 10 CVs.

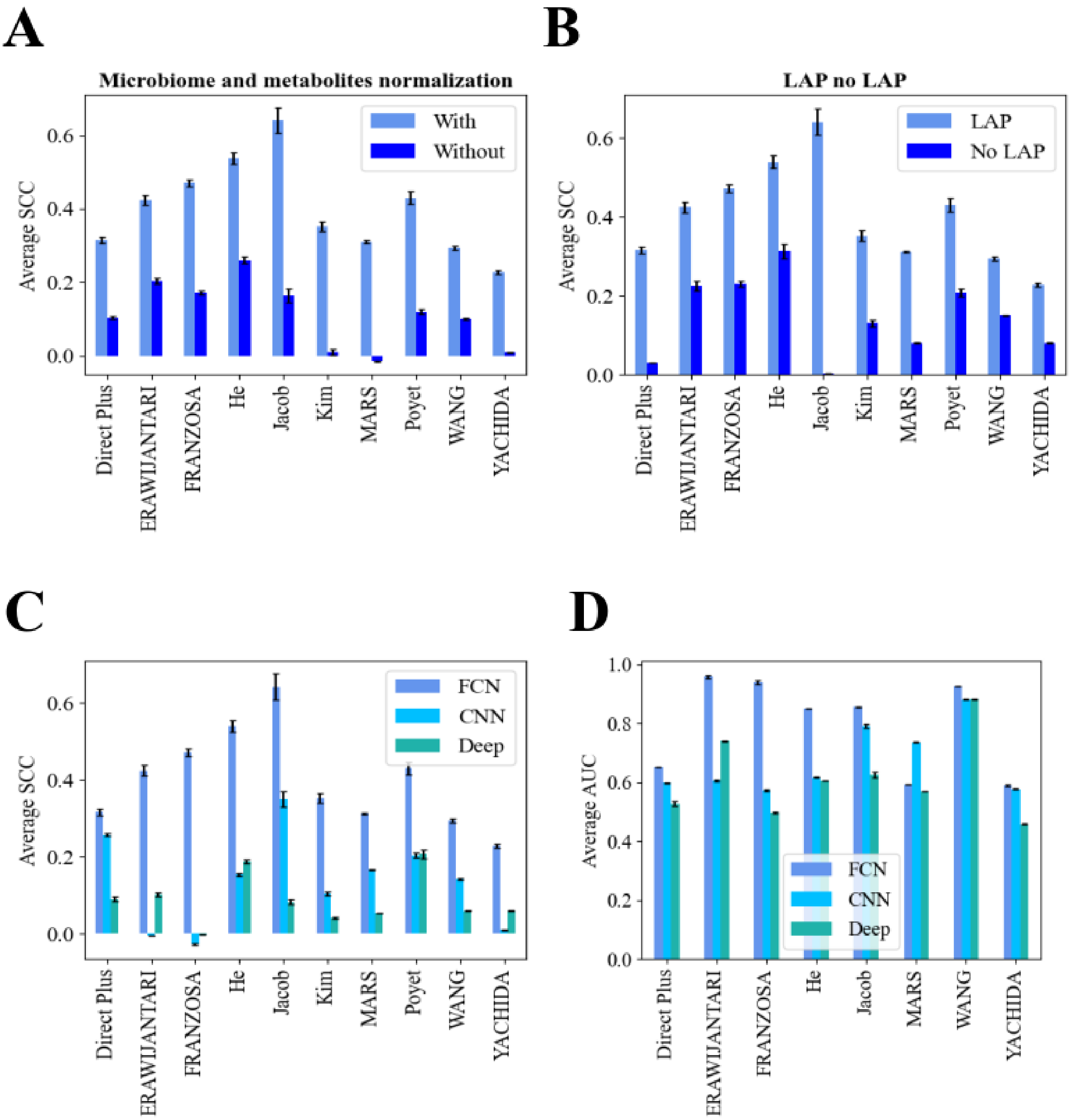

Figure 6: Comparison of various variants of LOCATE: **A.** Comparison of LOCATE with different normalization strategies for microbiome and metabolites (log and z-scoring) against the variant without normalization. **B.** Comparison of LOCATE with its second step of Low-Rank Approximation (LAP) against a regular encoder-decoder. **C.** Comparison of different methods of dimension reduction to create the intermediate representation Z (Fully Connected Network (FCN), 1D Convolutional Neural Network (1D-CNN), deep network with 5 CNN layers) in terms of metabolite prediction performance. **D.** Comparison of the same dimension reduction methods for the phenotype prediction performance. The black error bars represent the standard errors within the 10 cross-validation runs.

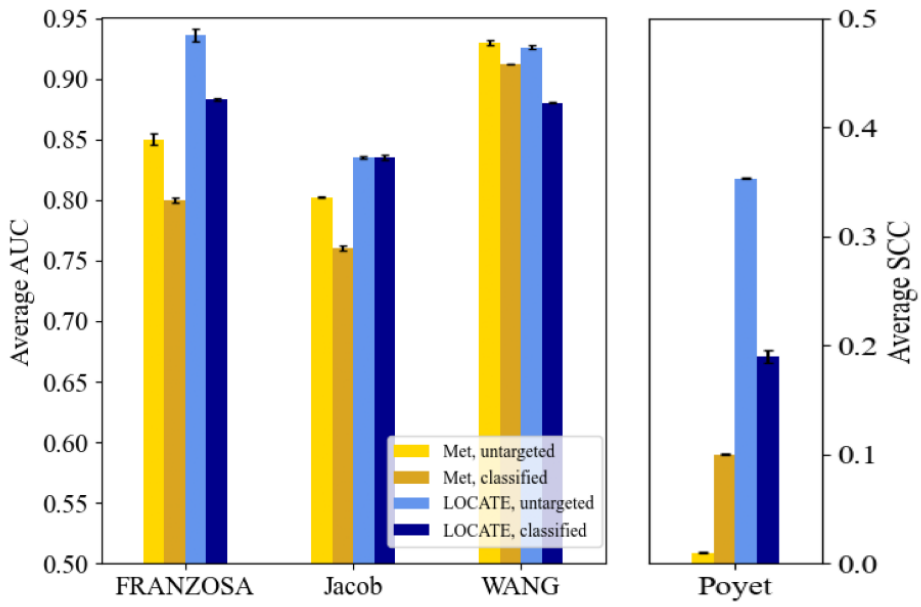

Figure 7: Host condition predictions based on targeted metabolites vs. untargeted metabolites. In each cohort with untargeted metabolites, the condition is predicted, once based on models that are trained only on classified metabolites (the dark bars), and once on all the metabolites including unclassified ones. LOCATE based on untargeted metabolites (light blue) outperforms all the other methods. The performance is measured as the average AUC (for binary phenotypes) and SCC for continuous phenotypes on a test set over 10 CVs. The black error bars represent the standard errors within the 10 CVs.

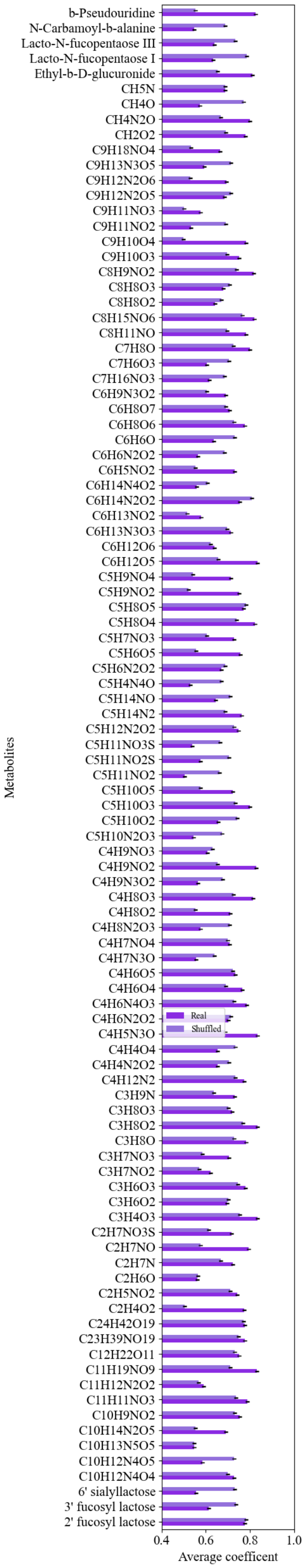

b-Pseudouridine
N-Carbamoyl-b-alanine
Lacto-N-fucopentaose III
Lacto-N-fucopentaose I
Ethyl-b-D-glucuronide
CH5N
CH4O
CH4N2O
CH2O2
C9H18NO4
C9H13N3O5
C9H12N2O6
C9H12N2O5
C9H11NO3
C9H11NO2
C9H10O4
C9H10O3
C8H9NO2
C8H8O3
C8H8O2
C8H15NO6
C8H11NO
C7H8O
C7H6O3
C7H16NO3
C6H9N3O2
C6H8O7
C6H8O6
C6H6O
C6H6N2O2
C6H5NO2
C6H14N4O2
C6H14N2O2
C6H13NO2
C6H13N3O3
C6H12O6
C6H12O5
C5H9NO4
C5H9NO2
C5H8O5
C5H8O4
C5H7NO3
C5H6O5
C5H6N2O2
C5H4N4O
C5H14NO
C5H14N2
C5H12N2O2
C5H11NO3S
C5H11NO2S
C5H11NO2
C5H10O5
C5H10O3
C5H10O2
C5H10N2O3
C4H9NO3
C4H9NO2
C4H9N3O2
C4H8O3
C4H8O2
C4H8N2O3
C4H7NO4
C4H7N3O
C4H6O5
C4H6O4
C4H6N4O3
C4H6N2O2
C4H5N3O
C4H4O4
C4H4N2O2
C4H12N2
C3H9N
C3H8O3
C3H8O2
C3H8O
C3H7NO3
C3H7NO2
C3H6O3
C3H6O2
C3H4O3
C2H7NO3S
C2H7NO
C2H7N
C2H6O
C2H5NO2
C2H4O2
C24H42O19
C23H39NO19
C12H22O11
C11H19NO9
C11H12N2O2
C11H11NO3
C10H9NO2
C10H14N2O5
C10H13N5O5
C10H12N4O5
C10H12N4O4
6' sialyllactose
3' fucosyl lactose
2' fucosyl lactose
Real
Shuffled
Metabolites
0.4
0.6
0.8
1.0
Average coefficent

Figure 8: Average coefficients of each metabolite in the real dataset (dark bar) and in the shuffled one (light bar). The black error bars are for the standard errors.

2 Supp. Mat. Tables

Table 1: Summary of current state-of-the-art methods

Model	Advantages	Disadvantages	Ref
**PRMT**	1. First framework.<br>2. Significant correlations between PRMT scores and relative abundances of selected environmental measurements.	1. Based on biological known networks.<br>2. Limited only to the KEGG database.<br>3. Performance is limited.<br>4. Not transferable (mixed datasets, between datasets).	[2]
**MIMOSA**	1. Gives information about the contribution of each taxon to the metabolites.<br>2. Succeeds in predicting relations in real and simulated datasets.<br>3. Freely available web server.	1. Based on biological known networks.<br>2. Limited only to the KEGG database.<br>3. Requires the original sequences data.<br>4. Performance is limited (worse than MelonnPan).<br>5. Not transferable (mixed datasets, between datasets).	[3, 4]
**Mangosteen**	1. Success in specific metabolites.	1. Based on biological known networks.<br>2. Limited only to the KEGG database.<br>3. Performance is limited (worse than MelonnPan and MIMOSA).<br>4. Not transferable (mixed datasets, between datasets).	[5]
**MelonnPan**	1. Best performance among existing state-of-the-art.<br>2. Quite a good definition for well-predicted metabolites.<br>3. Independent of previous biological knowledge.	1. Long-running times (run for each metabolite separately).<br>2. Cannot cope with all metabolites.<br>3. Sometimes returns the same prediction to all samples for specific metabolites.<br>4. Not transferable (mixed datasets, between datasets).	[6]
**MiMeNet**	1. Learning multiple metabolites simultaneously enables to find relations between the metabolites.<br>2. They claim it is better than existing state-of-the-art methods (PRMT, MelonnPan, and SparseNED).<br>3. Independent of previous biological knowledge.	1. Long-running time.<br>2. Performance is limited in the external test within a dataset.<br>3. Not transferable (mixed datasets, between datasets).	[7]
**SparseNED**	1. Learning multiple metabolites simultaneously enables to find relations between the metabolites.<br>2. Quite short running times.<br>3. Independent of previous biological knowledge.	1. Performance is limited within a dataset (worse than MelonnPan).<br>2. Not transferable (mixed datasets, between datasets).	[8]
**mNODE**	1. Outperforms existing methods in predicting the metabolomic profiles of human microbiomes and several environmental microbiomes.<br>2. Can incorporate dietary information for the prediction.<br>3. Independent of previous biological knowledge.	1. Performance is limited within a dataset.<br>2. Hyperparameters tuning as a mandatory step.<br>3. Long-running time.<br>4. Deep networks require a lot of training data.	[9]
**Khajeh et. al**	1. Learning multiple metabolites simultaneously enables to find relations between the metabolites.<br>2. Independent of previous biological knowledge.	1. Was tested on a single IBD cohort and a single task.<br>2. Autoencoders tend to need many samples for training.	[10]
**Multiview**	1. Achieves higher predictive phenotype accuracy than separate learning.<br>2. Powerful when the different views share some underlying relationships.	1. Falls short of creating a learnable connection between the microbiome and metabolites.	[11]
**Integrated Learner**	1. Achieves higher predictive phenotype accuracy than separate learning.<br>2. Enables uncertainty quantification in prediction.<br>3. Enables interval estimation for a variety of quantities.	1. Falls short of creating a learnable connection between the microbiome and metabolites.<br>2. Does not share information between layers during the first stage of learning.	[12]

Table 2: Datasets details

**Dataset**	**Cohort description**	**16S or WGS**	**N (species)**	**N (metabolites)**	**N (subjects)**		**N (samples)**		**Targeted / untargeted**	**Ref**
					Case	Control	Case	Control		
Direct Plus	18-month randomized clinical trial, we assigned 294 participants with abdominal obesity/dyslipidemia into healthy dietary guidelines (HDG), MED and green-MED weight-loss diet groups, all accompanied by physical activity.	16S	208	62	NA	294	NA	784	Targeted	[13]
Kim	Patients with advanced colorectal adenomas, colorectal cancer, and controls.	16S	85	462	138	102	138	102	Untargeted	[14]
He	Infants over several time points during the 1st year of life, either breast-fed, formula-fed, or experimental formula fed.	16S	47	120	NA	80	NA	277	Targeted	[15]
Jacob	Inflammatory bowel disease patients and their first degree (healthy) relatives.	16S	79	1307	36	54	36	54	Untargeted	[16]
Poyet	Longitudinal samples from healthy donors to the Broad Institute-OpenBiome Microbiome Library (BIO-ML).	16S	57	156	NA	83	NA	164	Untargeted	[17]
ERAWIJANTARI	Patients who underwent colonoscopy, half with a history of gastrectomyfor gastric cancer and no signs of gastric cancer recurrence.	WGS	12009	418	42	54	42	54	Targeted	[18]
FRANZOSA	Inflammatory bowel disease patients and controls (PRISM cohort + A validation cohort).	WGS	9154	8848	164	56	164	56	Untargeted	[19]
MARS	Longitudinal samples (over 6 months) from patients with Irritable Bowel Syndrome and controls.	WGS	4155	43	51	24	305	139	Targeted	[20]
WANG	Adults with end-stage renal disease (ESRD) and controls.	WGS	14950	276	220	67	220	67	Untargeted	[21]
YACHIDA	Patients who underwent colonoscopy, with findings from normal to stage 4 colorectal cancer.	WGS	16383	174	220	127	220	127	Targeted	[22]

Table 3: LOCATE's hyperparameters used

	**BGU**	**He**	**Jacob**	**Kim**	**Poyet**
**Activation function**	Tanh	elU	Tanh	Tanh	elU
**Dropout**	0.002	0.070	0.209	0.002	0.079
**Weight decay**	0.127	0.030	0.138	0.120	0.020
**Learning rate**	0.001	0.001	0.05	0.001	0.001
**Number of neurons layer1**	90	20	20	90	20
**Number of neurons layer 2**	80	10	30	80	10
**Representation size**	10	10	10	10	10
**Optimizer**	Adam	Adam	Adam	Adam	Adam
**Max epochs**	1000	1000	1000	1000	1000

Table 4: Metadata of each cohort

**Dataset**	**Metadata used**
Direct Plus	Diet, sex, height
He	Diet, age, sex
Jacob	Sex, age, pedigree
Poyet	Travel abroad last year, seasonal Pollen allergy, weight, height, BMI, country of birth, sex, relationship status, pet allergy, diet, age
Kim	Age, sex, race, smoking history
ERAWIJANTARI	Smoking status, lung cancer, drinking status, breast cancer, glucose, liver cancer, total cholesterol, diabetes med, analgesic, anticoagulant, gastric acid medication, high blood pressure, uterine cancer, sex, alcohol consumption, age
FRANZOSA	Age, antibiotic, immunosuppressant, mesalamine, steroids,
WANG	Age, BMI, Creatinine , Urea, eGFR, sex
MARS	Age, BMI, sex, antibiotics
YACHIDA	Age, sex, BMI, alcohol

Table 5: WGS 4 different clusters cross-datasets WGS

Pair	Cluster num	Color
s__Pauljensenia turicensis-C5H11NO2	1	light grey
s__Collinsella sp900551195-C6H13NO2	1	light grey
s__Collinsella sp900551605-C6H13NO2	1	light grey
s__Collinsella sp900759435-C4H4N2O2	1	light grey
s__Eggerthella lenta-C6H13NO2	1	light grey
s__Eggerthella sp014287365-C6H13NO2	1	light grey
s__Prevotella sp000431975-C4H4N2O2	1	light grey
s__Alistipes sp002428825-C4H4N2O2	1	light grey
s__Alistipes sp900021155-C4H4N2O2	1	light grey
s__Tidjanibacter inops_A-C4H4N2O2	1	light grey
s__Confluentibacter sp003258355-C5H4N4O	1	light grey
s__Clostridium saudiense-C6H13NO2	1	light grey
s__Clostridium sp900543325-C6H13NO2	1	light grey
s__Acetatifactor sp002431915-C4H4N2O2	1	light grey
s__Acetatifactor sp900771995-C4H4N2O2	1	light grey
s__Acetatifactor sp900772845-C4H4N2O2	1	light grey
s__Coprococcus sp900548315-C4H4N2O2	1	light grey
s__Lachnospira sp900547255-C4H4N2O2	1	light grey
s__UBA11774 sp003507655-C3H5O3-	1	light grey
s__UBA7182 sp002491115-C4H4N2O2	1	light grey
s__Acutalibacter sp009936035-C4H4N2O2	1	light grey
s__Acutalibacter sp900543305-C4H4N2O2	1	light grey
s__Ruminococcus_E sp900315195-C6H14N2O2	1	light grey
s__Ruminococcus_E sp902797655-C6H14N2O2	1	light grey
s__UBA737 sp900554525-C4H4N2O2	1	light grey
s__CAG-170 sp000432135-C4H4N2O2	1	light grey
s__Dysosmobacter sp900752075-C4H4N2O2	1	light grey
s__UBA5446 sp900543085-C4H4N2O2<br>1	1<br>light grey	light s__Ruminococcus sp900540005-C4H4N2O2
s__CAG-145 sp900545135-C4H4N2O2	1	light grey
s__Emergencia sp900551775-C4H4N2O2	1	light grey
s__NSJ-50 sp014385105-C4H4N2O2	1	light grey
s__UBA2862 sp902790525-C3H7NO2	1	light grey
s__Christensenella massiliensis-C26H43NO6	1	light grey
s__UBA2897 sp002350105-C6H14N2O2	1	light grey
s__Fusobacterium_A sp900015295-C3H7NO2	1	light grey
s__D16-34 sp009911635-C3H5O2-	2	dark grey
s__Alistipes sp002428825-C5H4N4O	2	dark grey
s__Alistipes sp900549305-C5H4N4O	2	dark grey
s__Parabacteroides sp011038785-C4H4N2O2	2	dark grey
s__RC9 sp900546445-C5H4N4O	2	dark grey
s__Streptococcus hyointestinalis-C6H13NO2	2	dark grey
s__Streptococcus parasanguinis_A-C6H13NO2	2	dark grey
s__Streptococcus parasanguinis_B-C6H13NO2	2	dark grey
s__Streptococcus parasanguinis_C-C6H13NO2	2	dark grey

Table 5: WGS 4 different clusters cross-datasets WGS

Pair	Cluster num	Color
s__Streptococcus parasanguinis_D-C6H13NO2	2	dark grey
s__Streptococcus sp000314795-C6H13NO2	2	dark grey
s__Streptococcus sp000448565-C6H13NO2	2	dark grey
s__Streptococcus sp900543065-C6H13NO2	2	dark grey
s__Streptococcus sp900766505-C6H13NO2	2	dark grey
s__UBA9502 sp004554205-C5H4N4O	2	dark grey
s__NSJ-32 sp014384895-C5H4N4O	2	dark grey
s__CAG-110 sp003525905-C5H4N4O	2	dark grey
s__CAG-83 sp900545585-C5H4N4O	2	dark grey
s__Dysosmobacter sp001916835-C5H4N4O	2	dark grey
s__ER4 sp900552015-C5H4N4O	2	dark grey
s__Flavonifractor sp900549795-C5H4N4O	2	dark grey
s__HGM12998 sp900756495-C5H4N4O	2	dark grey
s__Intestinimonas butyriciproducens-C5H4N4O	2	dark grey
s__Intestinimonas massiliensis-C5H4N4O	2	dark grey
s__UBA3855 sp902783005-C3H5O2-	2	dark grey
s__UMGS1889 sp900556055-C5H4N4O	2	dark grey
s__Emergencia sp900551775-C26H43NO6	2	dark grey
s__Phil1 sp001940855-C5H4N4O	2	dark grey
s__UMGS692 sp900544545-C3H5O2-	2	dark grey
s__Firm-10 sp001603025-C3H5O2-	2	dark grey
s__HGM11575 sp002068815-C3H5O2-	2	dark grey
s__UBA2862 sp900315585-C3H5O2-	2	dark grey
s__UBA2862 sp900318045-C3H5O2-	2	dark grey
s__UBA2862 sp902798105-C3H5O2-	2	dark grey
s__QALW01 sp003150515-C26H43NO6	2	dark grey
s__Akkermansia sp004167605-C3H5O2-	2	dark grey
s__Porphyromonas sp900539155-C5H11NO2	3	dim grey
s__Alistipes putredinis-C5H11NO2	3	dim grey
s__Alistipes senegalensis-C5H11NO2	3	dim grey
s__Alistipes shahii-C5H11NO2	3	dim grey
s__Alistipes sp900021155-C5H11NO2	3	dim grey
s__Alistipes sp900541585-C5H11NO2	3	dim grey
s__Alistipes_A indistinctus-C5H11NO2	3	dim grey
s__UBA940 sp900768115-C5H11NO2	3	dim grey
s__W3P20-009 sp004552385-C5H11NO2	3	dim grey
s__NSJ-32 sp014384895-C5H11NO2	3	dim grey
s__Acutalibacter timonensis-C5H11NO2	3	dim grey
s__NSJ-40 sp014384705-C5H11NO2	3	dim grey
s__UMGS856 sp900760305-C5H11NO2	3	dim grey
s__CAG-390 sp000437015-C5H11NO2	3	dim grey
s__CAG-390 sp900753295-C5H11NO2	3	dim grey
s__CAG-841 sp000437375-C5H11NO2	3	dim grey
s__HGM12650 sp900761725-C5H11NO2	3	dim grey
s__UMGS1002 sp900547565-C5H11NO2	3	dim grey

Table 5: WGS 4 different clusters cross-datasets WGS

Pair	Cluster num	Color
s__UMGS1696 sp900763885-C5H11NO2	3	dim grey
s__SFLA01 sp004553575-C5H11NO2	3	dim grey
s__UCG-010 sp900754535-C5H11NO2	3	dim grey
s__CAG-103 sp900317855-C5H11NO2	3	dim grey
s__CAG-110 sp900546415-C5H11NO2	3	dim grey
s__CAG-110 sp900548795-C5H11NO2	3	dim grey
s__CAG-110 sp900551495-C5H11NO2	3	dim grey
s__CAG-110 sp900554625-C5H11NO2	3	dim grey
s__CAG-110 sp900769995-C5H11NO2	3	dim grey
s__CAG-170 sp000436735-C5H11NO2	3	dim grey
s__CAG-170 sp002437575-C5H11NO2	3	dim grey
s__CAG-170 sp900548625-C5H11NO2	3	dim grey
s__CAG-83 sp900548615-C5H11NO2	3	dim grey
s__Flavonifractor massiliensis_A-C5H11NO2	3	dim grey
s__Marseille-P3106 sp900169975-C5H11NO2	3	dim grey
s__Pseudoflavonifractor sp900079765-C5H11NO2	3	dim grey
s__Anaerotruncus rubiinfantis-C5H11NO2	3	dim grey
s__Anaerotruncus sp014385085-C5H11NO2	3	dim grey
s__Massilimaliae massiliensis-C5H11NO2	3	dim grey
s__UBA1394 sp002305725-C5H11NO2	3	dim grey
s__BX12 sp009911365-C5H11NO2	3	dim grey
s__CAG-145 sp900545135-C5H11NO2	3	dim grey
s__CAG-238 sp002439735-C5H11NO2	3	dim grey
s__Phil1 sp001940855-C5H11NO2	3	dim grey
s__Firm-11 sp900548145-C5H11NO2	3	dim grey
s__SFFS01 sp004557805-C5H11NO2	3	dim grey
s__NSJ-63 sp014384805-C5H11NO2	3	dim grey
s__Alistipes onderdonkii-C6H13NO2	4	k
s__Alistipes sp002358415-C6H13NO2	4	k
s__Alistipes sp002362235-C6H13NO2	4	k
s__Alistipes sp900290115-C6H13NO2	4	k
s__Alistipes sp900541585-C6H13NO2	4	k
s__Alistipes sp902388705-C6H13NO2	4	k
s__UMGS2068 sp900769635-C6H13NO2	4	k
s__Anaerofustis stercorihominis-C6H13NO2	4	k
s__Blautia_A luti-C3H5O3-	4	k
s__Blautia_A sp900540785-C4H4N2O2	4	k
s__CAG-317 sp011960265-C4H4N2O2	4	k
s__HGM11523 sp900756545-C6H13NO2	4	k
s__Agathobaculum sp900291975-C6H13NO2	4	k
s__Lawsonibacter sp900066825-C6H13NO2	4	k
s__Lawsonibacter sp900764755-C6H13NO2	4	k
s__Anaerotruncus colihominis-C6H13NO2	4	k
s__UBA1409 sp002305045-C24H40O5	4	k
s__BX12 sp009911365-C6H13NO2	4	k

Table 5: WGS 4 different clusters cross-datasets WGS

Pair	Cluster num	Color
s__CAG-145 sp900545135-C6H13NO2	4	k
s__Emergencia sp009935805-C6H13NO2	4	k
s__Mogibacterium timidum-C4H4N2O2	4	k
s__RUG100 sp900315555-C6H13NO2	4	k
s__Fusobacterium_A varium-C4H4N2O2	4	k

Table 6: Acronym table

**Acronym**	**Meaning**
LOCATE	Latent variables Of miCrobiome And meTabolites rElations
ML	Machine Learning
SCFA	Short Chain Fatty Acids
T1D	Type 1 Diabetes
IBD	Inflammatory bowel disease
T2D	Type 2 Diabetes
DNN	Deep Neural Networks
CNN	Convolutional Neural Networks
PRMT	Predicted Relative Metabolomic Turnover
MIMOSA	Model-based Integration of Metabolite Observations and Species Abundance
MLPNN	Multiple-layer Perceptron Neural network
WGS	Whole Genome Shotgun Sequencing
HDG	Healthy Dietary Guidelines
MRS	Magnetic Resonance Spectroscopy
DSC	Deep Subcutaneous
SSC	Superficial Subcutaneous
VAT	Visceral Adipose Tissue
CD	Crohn's Disease
UC	Ulcerative Colitis
ESRD	End-Stage Renal Disease
NMF	Non Negative Matrix Factorization
NNI	Neural Network Intelligence
MSE	Mean Square Error
SCC	Spearman Correlation Coefficient
AUC	Area Under the ROC Curve
CCA	Canonical-Correlation Analysis
SVD	Singular Value Decomposition
CRC	Colorectal Cancer

Table 7: Clustering components of the metadata features in Fig. 5 B, C,D.

Dataset	Cluster	Cluster components
He	Diet	Continuous
	Age	Continuous
	Sex	Continuous
Jacob	Sex	Continuous
	Age	Continuous
	Pedigree	Continuous
Poyet	Travel abroad last year	Bosnia, Canada, China, Costa Rica,Croatia, Europe, Iceland, Japan, India, South KoreaIreland and England, Italy, France, Spain, Poland, South AfricaPuerto Rico, Mexico, Caribbean area, Portugal, Switzerland, No
	Seasonal Pollen allergy	Hay fever like symptoms, in WA state, finished 2 years ago, Mild dust allergy, Mild pollen, No, Pollen, Seasonal, runny nose/stuffy sinuses, Yes, Yes (mold)
	Weight	Continuous
	Height	Continuous
	BMI	Continuous
	Country birth	Bosnia, Canada, USA
	Sex	Female, male
	Relationship status	Dating, Married, Relationship, Single
	Pet allergy	Cat, dog, No
	Diet	Omnivore, Vegetarian,
	Age	Continuous

Table 8: Clustering components of Fig. 5 F, G and H. Each cluster is represented by 2 colors of its 2 first dimensions.

**Dataset**	**Cluster**	**Cluster components**
ERAWIJANTARI	Age	Continuous
	Alcohol consumption	Continuous
	Analgesic	Binary
	Anticoagulant	Binary
	Breast cancer	Binary
	DiabetesMed	Binary
	Drinking status	Drink, Not Drinking, Stop Drinking, Unknown
	Gastric acid medication	Binary
	Gender	Male, female
	Glucose	Continuous
	High blood pressure	Binary
	Liver cancer	Binary
	Lung cancer	Binary
	Smoking status	Smoke, Not smoking, Stop smoking, Unknown
	Total cholesterol	Continuous
	Uterine cancer	Binary
FRANZOSA	Age	Continuous
	Antibiotic	Binary
	Immunosuppressant	Binary
	Steroids	Binary
	Mesalamine	Binary
WANG	Age	Continuous
	Gender	Male, female
	BMI	Continuous
	Urea	Continuous
	Creatinine	Continuous
	eGFR	Continuous

References

[1] Roktaek Lim, Josephine Jill T. Cabatbat, Thomas L.P. Martin, Haneul Kim, Seunghyeon Kim, Jaeyun Sung, Cheol-Min Ghim, and Pan-Jun Kim. Large-scale metabolic interaction network of the mouse and human gut microbiota. *Scientific Data*, 7(1):1–8, 2020.

[2] Peter E. Larsen, Frank R. Collart, Dawn Field, Folker Meyer, Kevin P. Keegan, Christopher S. Henry, John McGrath, John Quinn, and Jack A. Gilbert. Predicted relative metabolomic turnover (PRMT): determining metabolic turnover from a coastal marine metagenomic dataset. *Microbial Informatics and Experimentation*, 1(1):1–11, 2011.

[3] Cecilia Noecker, Alexander Eng, Sujatha Srinivasan, Casey M. Theriot, Vincent B. Young, Janet K. Jansson, David N. Fredricks, and Elhanan Borenstein. Metabolic model-based integration of microbiome taxonomic and metabolomic profiles elucidates mechanistic links between ecological and metabolic variation. *MSystems*, 1(1):e00013–15, 2016.

[4] Cecilia Noecker, Alexander Eng, Efrat Muller, and Elhanan Borenstein. Mimosa2: a metabolic network-based tool for inferring mechanism-supported relationships in microbiome-metabolome data. *Bioinformatics*, 38(6):1615–1623, 2022.

[5] Xiaochen Yin, Tomer Altman, Erica Rutherford, Kiana A. West, Yonggan Wu, Jinlyung Choi, Paul L. Beck, Gilaad G. Kaplan, Karim Dabbagh, Todd Z. DeSantis, et al. A comparative evaluation of tools to predict metabolite profiles from microbiome sequencing data. *Frontiers in Microbiology*, 11:3132, 2020.

[6] Himel Mallick, Eric A. Franzosa, Lauren J. Mclver, Soumya Banerjee, Alexandra Sirota-Madi, Aleksandar D. Kostic, Clary B. Clish, Hera Vlamakis, Ramnik J. Xavier, and Curtis Huttenhower. Predictive metabolomic profiling of microbial communities using amplicon or metagenomic sequences. *Nature Communications*, 10(1):1–11, 2019.

[7] Derek Reiman, Brian T. Layden, and Yang Dai. Mimenet: Exploring microbiome-metabolome relationships using neural networks. *PLOS Computational Biology*, 17(5):e1009021, 2021.

[8] Vuong Le, Thomas P. Quinn, Truyen Tran, and Svetha Venkatesh. Deep in the bowel: highly interpretable neural encoder-decoder networks predict gut metabolites from gut microbiome. *BMC Genomics*, 21(4):1–15, 2020.

[9] Tong Wang, Xu-Wen Wang, Kathleen A Lee-Sarwar, Augusto A Litonjua, Scott T Weiss, Yizhou Sun, Sergei Maslov, and Yang-Yu Liu. Predicting metabolomic profiles from microbial composition through neural ordinary differential equations. *Nature Machine Intelligence*, 5(3):284–293, 2023.

[10] Tina Khajeh, Derek Reiman, Ryan Morley, and Yang Dai. Integrating microbiome and metabolome data for host disease prediction via deep neural networks. In *2021 IEEE EMBS International Conference on Biomedical and Health Informatics (BHI)*, pages 1–4. IEEE, 2021.

[11] Daisy Yi Ding, Shuangning Li, Balasubramanian Narasimhan, and Robert Tibshirani. Cooperative learning for multiview analysis. *Proceedings of the National Academy of Sciences*, 119(38):e2202113119, 2022.

[12] Himel Mallick, Anupreet Porwal, Satabdi Saha, Piyali Basak, Vladimir Svetnik, and Erina Paul. An integrated bayesian framework for multi-omics prediction and classification. *bioRxiv*, pages 2022–11, 2022.

[13] Anat Yaskolka Meir, Ehud Rinott, Gal Tsaban, Hila Zelicha, Alon Kaplan, Philip Rosen, Ilan Shelef, Ilan Youngster, Aryeh Shalev, Matthias Blüher, et al. Effect of green-mediterranean diet on intrahepatic fat: the DIRECT PLUS randomised controlled trial. *Gut*, 70(11):2085–2095, 2021.

[14] Minsuk Kim, Emily Vogtmann, David A. Ahlquist, Mary E. Devens, John B Kisiel, William R. Taylor, Bryan A. White, Vanessa L. Hale, Jaeyun Sung, Nicholas Chia, et al. Fecal metabolomic signatures in colorectal adenoma patients are associated with gut microbiota and early events of colorectal cancer pathogenesis. *MBio*, 11(1):e03186–19, 2020.

[15] Xuan He, Mariana Parenti, Tove Grip, Bo Lönnerdal, Niklas Timby, Magnus Domellöf, Olle Hernell, and Carolyn M Slupsky. Fecal microbiome and metabolome of infants fed bovine mfgm supplemented formula or standard formula with breast-fed infants as reference: a randomized controlled trial. *Scientific Reports*, 9(1):1–14, 2019.

[16] Jonathan P Jacobs, Maryam Goudarzi, Namita Singh, Maomeng Tong, Ian H McHardy, Paul Ruegger, Miro Asadourian, Bo-Hyun Moon, Allyson Ayson, James Borneman, et al. A disease-associated microbial and metabolomics state in relatives of pediatric inflammatory bowel disease patients. *Cellular and Molecular Gastroenterology and Hepatology*, 2(6):750–766, 2016.

[17] Mathilde Poyet, Mathieu Groussin, Sean M. Gibbons, J. Avila-Pacheco, Xiaofang Jiang, Sean M. Kearney, A.R. Perrotta, B. Berdy, S. Zhao, T.D. Lieberman, et al. A library of human gut bacterial isolates paired with longitudinal multiomics data enables mechanistic microbiome research. *Nature Medicine*, 25(9):1442–1452, 2019.

[18] Pande Putu Erawijantari, Sayaka Mizutani, Hirotsugu Shiroma, Satoshi Shiba, Takeshi Nakajima, Taku Sakamoto, Yutaka Saito, Shinji Fukuda, Shinichi Yachida, and Takuji Yamada. Influence of gastrectomy for gastric cancer treatment on faecal microbiome and metabolome profiles. *Gut*, 69(8):1404–1415, 2020.

[19] Eric A. Franzosa, Alexandra Sirota-Madi, Julian Avila-Pacheco, Nadine Fornelos, Henry J. Haiser, Stefan Reinker, Tommi Vatanen, A Brantley Hall, Himel Mallick, Lauren J. McIver, et al. Gut microbiome structure and metabolic activity in inflammatory bowel disease. *Nature Microbiology*, 4(2):293–305, 2019.

[20] Ruben AT Mars, Yi Yang, Tonya Ward, Mo Houtti, Sambhawa Priya, Heather R Lekatz, Xiaojia Tang, Zhifu Sun, Krishna R. Kalari, Tal Korem, et al. Longitudinal multi-omics reveals subset-specific mechanisms underlying irritable bowel syndrome. *Cell*, 182(6):1460–1473, 2020.

[21] Xifan Wang, Songtao Yang, Shenghui Li, Liang Zhao, Yanling Hao, Junjie Qin, Lian Zhang, Chengying Zhang, Weijing Bian, LI Zuo, et al. Aberrant gut microbiota alters host metabolome and impacts renal failure in humans and rodents. *Gut*, 69(12):2131–2142, 2020.

[22] Shinichi Yachida, Sayaka Mizutani, Hirotsugu Shiroma, Satoshi Shiba, Takeshi Nakajima, Taku Sakamoto, Hikaru Watanabe, Keigo Masuda, Yuichiro Nishimoto, Masaru Kubo, et al. Metagenomic and metabolomic analyses reveal distinct stage-specific phenotypes of the gut microbiota in colorectal cancer. *Nature Medicine*, 25(6):968–976, 2019.
